# Merkel cell polyomavirus small T antigen is a viral transcription activator that is essential for viral genome maintenance

**DOI:** 10.1371/journal.ppat.1011039

**Published:** 2022-12-27

**Authors:** Kyle Rapchak, Shiva D. Yagobian, Jackson Moore, Michelle Khattri, Masahiro Shuda

**Affiliations:** 1 Cancer Virology Program, University of Pittsburgh Medical Center (UPMC) Hillman Cancer Center, Pittsburgh, Pennsylvania, United States of America; 2 Department of Microbiology and Molecular Genetics, University of Pittsburgh School of Medicine, Pittsburgh, Pennsylvania, United States of America; Brown University, UNITED STATES

## Abstract

Merkel cell polyomavirus (MCV) is a small DNA tumor virus that persists in human skin and causes Merkel cell carcinoma (MCC) in immunocompromised individuals. The multi-functional protein MCV small T (sT) activates viral DNA replication by stabilizing large T (LT) and promotes cell transformation through the LT stabilization domain (LTSD). Using MCVΔsT, a mutant MCV clone that ablates sT, we investigated the role of sT in MCV genome maintenance. sT was dispensable for initiation of viral DNA replication, but essential for maintenance of the MCV genome and activation of viral early and late gene expression for progression of the viral lifecycle. Furthermore, in phenotype rescue studies, exogenous sT activated viral DNA replication and mRNA expression in MCVΔsT through the LTSD. While exogenous LT expression, which mimics LT stabilization, increased viral DNA replication, it did not activate viral mRNA expression. After cataloging transcriptional regulator proteins by proximity-based MCV sT-host protein interaction analysis, we validated LTSD-dependent sT interaction with four transcriptional regulators: Cux1, c-Jun, BRD9, and CBP. Functional studies revealed Cux1 and c-Jun as negative regulators, and CBP and BRD9 as positive regulators of MCV transcription. CBP inhibitor A-485 suppressed sT-induced viral gene activation in replicating MCVΔsT and inhibited early gene expression in MCV-integrated MCC cells. These results suggest that sT promotes viral lifecycle progression by activating mRNA expression and capsid protein production through interaction with the transcriptional regulators. This activity is essential for MCV genome maintenance, suggesting a critical role of sT in MCV persistence and MCC carcinogenesis.

## Author summary

Merkel cell polyomavirus (MCV) is a nearly ubiquitous small DNA virus that persists in human skin and causes Merkel cell carcinoma (MCC) in immunocompromised individuals. The MCV small T (sT) protein is known to indirectly activate viral replication by stabilizing large T (LT), and to promote malignant transformation through the LT stabilization domain (LTSD). However, the function of sT in the native MCV lifecycle remains unknown. This report reveals additional, critical roles of sT that drive progression of the MCV lifecycle. sT supports viral genome maintenance by regulating viral transcription through interaction with host transcriptional regulators including CBP, which activates viral gene expression in both episomal MCV and integrated MCV in MCC tumors. This research suggests that MCV sT is an essential factor for viral persistence and thus for MCC carcinogenesis.

## Introduction

Merkel cell polyomavirus (MCV) is an etiological agent for Merkel cell carcinoma (MCC), a rare neuroendocrine skin cancer associated with immunosuppression [1]. MCV is a ubiquitous virus that establishes lifelong infection. This virus persistently infects skin cells and is chronically shed from healthy human skin, suggesting that the virus productively replicates in immunocompetent individuals [2]. Since MCV infection induces cancer through persistent infection, understanding the molecular mechanism of MCV persistence may provide important clues towards the prevention of MCC. Yet, how this common virus persists in skin cells and maintains the viral genome remains unknown.

Like other polyomaviruses, the MCV T antigen gene encodes large T (LT) and small T (sT) proteins [3]. MCC tumors express sT along with a helicase-truncated form of the LT protein that is required for MCC cancer cell proliferation, consistent with the characterization of MCV T antigen serving as a viral oncogene [4]. Specifically, MCV sT is the first known polyomavirus sT protein that is sufficient to transform immortalized rodent fibroblasts in a PP2A-independent mechanism, whereas SV40 sT assists cell transformation by targeting PP2A when expressed together with LT [5]. MCV sT also possesses a unique function that activates viral DNA replication by stabilizing the LT replicase protein through inhibition of LT turnover mediated by SCF^Fbw7^ [6], although the involvement of SCF^Fbw7^ in sT-mediated LT stabilization remains debated [7]. Interestingly, these transformation and replication activation functions are mediated by a single domain of sT called the large T stabilization domain (LTSD), which spans amino acids (aa) 91–95.

The role of sT in the viral lifecycle and viral replication has yet to be studied extensively in other polyomaviruses. In an SV40 study, sT was found to be dispensable for the initiation of DNA replication when an sT-deletion mutant was infected into susceptible monkey cells, yet this sT deletion resulted in decreased viral DNA after 72 h [8]. Another SV40 sT study with semi-permissive human mesothelial cells found no difference between sT-deletion and wild type viruses at day 2 post-infection, but the sT deletion virus lost a detectable genome at 3 weeks post-infection, while the wild type SV40 infected cells retained high copies of the viral genome [9]. Although this phenotype was rescued by wild type SV40 sT, it could not be rescued by the sT mutant, which cannot bind to PP2A, suggesting that SV40 sT regulates long-term viral genome maintenance by inhibiting PP2A [10]. Furthermore, human JC polyomavirus (JCV) has been shown to require sT, as deletion of sT from the JCV genome ablates replication in primary fetal glial cells even at 7 days post-infection. Like SV40, JCV requires the PP2A-targeting domain for maintenance of the viral genome [10]. Taken together, these studies indicate a critical role of sT and its interaction with PP2A in viral genome maintenance and persistence.

MCV LT is a viral replication protein that is targeted by multiple host SCF E3 ligases. The LT protein level is sensitively regulated by viral sT and by the host cellular environment, including growth factors and nutrition levels [11,12]. For instance, the LT protein level is under negative control by mTOR-SCF^Skp2^, and thus growth factor depletion and mTOR inhibition lead to LT stabilization for active replication, as demonstrated by MCV origin plasmid replication (MCV replicon) assays with co-expression of MCV LT and/or sT [13,14]. These data suggest that MCV persistence may be regulated by sT and the cellular environment in a protein-mediated latency mechanism that controls LT replicase protein levels [12]. It should be noted that the majority of the aforementioned sT functions were revealed by MCV replicon assays that exclusively analyze the process of LT-dependent DNA replication. The role of native sT in the MCV life cycle remains unclear.

A reverse genetics approach with a circularized MCV genome allows for the study of the importance of sT in the MCV lifecycle [15–18]. In this study, we generated MCVΔsT, a mutant MCV genome with a deletion of sT, and found that MCVΔsT cannot maintain its genome as efficiently as wild type MCV (MCV_WT_), yet it is still capable of initiating replication. The defective MCVΔsT genome maintenance was associated with a significant decrease in viral DNA copy number, early and late mRNA expression, and MCV LT and VP1 protein expression in both MCV-transfected 293 cells and an MCV-infected human fibroblast cell line. Using MCVΔsT for phenotype rescue experiments with exogenously expressed sT and LT, we demonstrate that, in addition to its anticipated effect on viral DNA replication, MCV sT activates viral early and late mRNA expression. This activation of early and late mRNA expression occurs via the LTSD domain and not the PP2A binding domain as in SV40. Furthermore, a replication-defective mutation in the non-coding control region (NCCR) together with DNA replication inhibition significantly reduced activation of viral mRNA by sT, suggesting the importance of concurrent viral DNA replication in viral gene transcription.

Proximity-based biotin ligation assays with MCV sT_WT_ and the sT_LTSD_ mutant revealed host cell interaction candidates that bind to sT through the LTSD. The three functional groups of cellular proteins that localized closest to sT were associated with nucleic acid metabolism, metabolite interconversion enzymes, and cytoskeletal proteins. Of the transcriptional regulators identified by the proximity-based interaction analysis, we confirmed that transcriptional regulators Cux1, c-Jun, CBP, and BRD9 bind to MCV sT. Loss-of-function studies revealed that Cux1 and c-Jun are negative regulators of viral transcription, while CBP and BRD9 are positive regulators. A CBP-specific inhibitor decreased sT-induced early and late gene expression in episomal viral DNA and suppressed early gene expression in the integrated viral DNA in MCV-positive (+) MCC cells.

In this study, we sought to define the role of sT in native MCV replication using a sT-null MCV mutant. The results suggest that sT function, which activates viral early and late gene expression through interaction with host transcription regulators, is required for MCV genome maintenance and essential for progression of the MCV lifecycle. This finding led us to investigate the molecular mechanisms by which sT controls viral early and late gene expression in episomal MCV and T antigen gene expression in MCV+MCC, which are critical for viral persistence and Merkel cell carcinogenesis.

## Results

### sT deletion from the MCV genome results in a loss of MCV propagation in transfected cells

**Fig 1:**
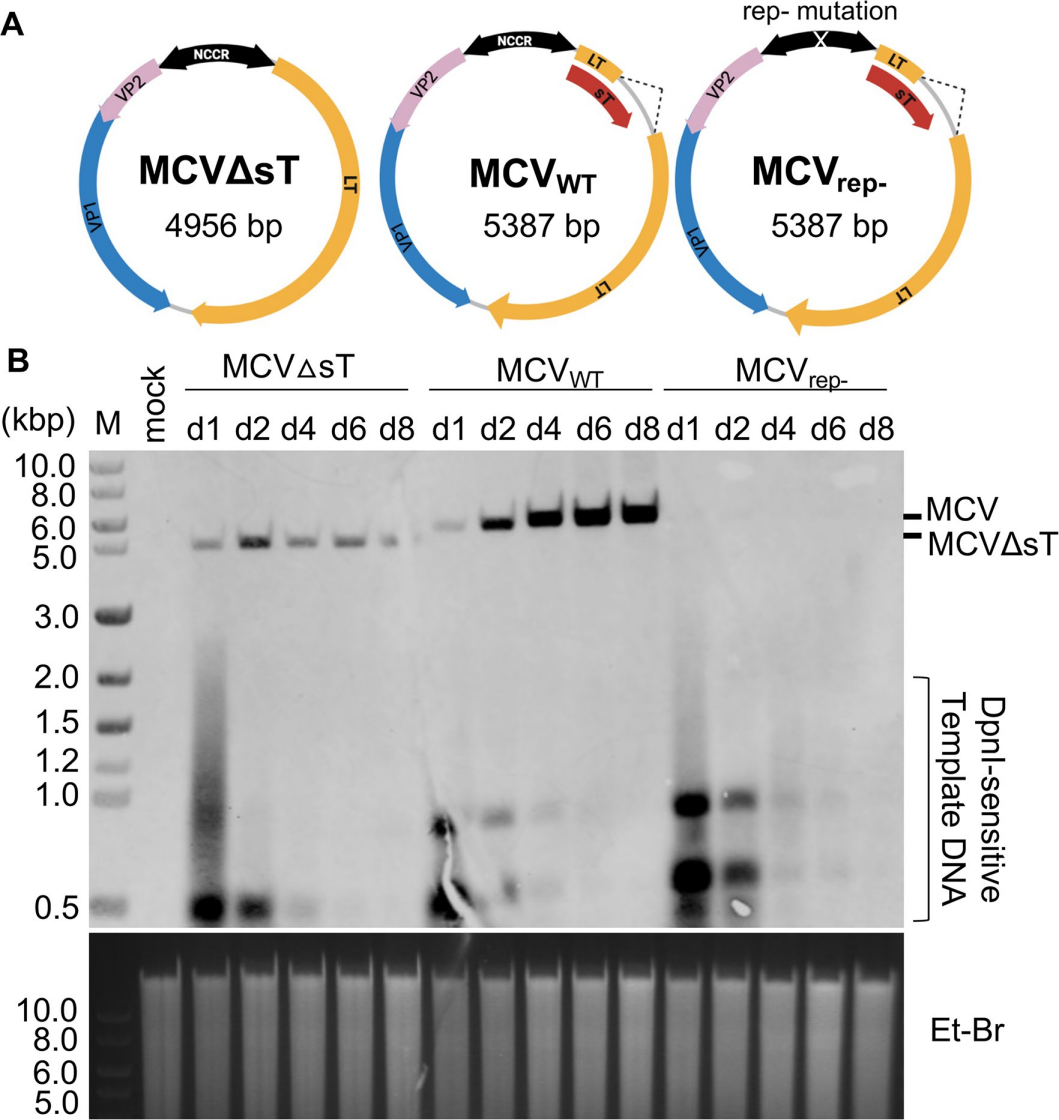
MCVΔsT cannot maintain its genome in transfected 293 cells. **(A)** MCVΔsT with a deletion of sT-specific exon (dotted line in wild type MCV), wild type MCV (MCV_WT_), and MCV_rep-_ with an NCCR mutation that ablates LT binding to the origin. **(B)** 293 cells transfected with MCV_WT_, MCVΔsT, and MCV_rep-_ were harvested at various time points up to 8 days p.t. Extracted genomic DNA was digested with EcoRI and DpnI and subjected to Southern blot with an MCV probe. Ethidium bromide (Et-Br) staining was used as a loading control (bottom image). M denotes DNA size marker.

Although a co-expression study of MCV sT and LT proteins revealed that sT activates MCV DNA replication through LT stabilization, the breadth of function of native sT expressed from the viral genome remains unknown. Thus, we exploited the native circular MCV genome, which was prepared by re-circularization of the previously reported MCV-HF clone [16]. We generated an sT null mutant (MCVΔsT) by deleting the entire LT intron that contains the sT-specific exon (nucleotide position 430–860, GeneBank accession no. JF813003). We also used a previously reported replication-incompetent MCV (MCV_rep-_) with an MCC-derived point mutation in the NCCR [13] (**Fig 1A**). We anticipated that only sT expression would be ablated in MCVΔsT, since the non-sT functional elements are not mapped within the deleted sT exon.

First, we examined replication activity by transfecting re-circularized MCV genomes into 293 cells. To avoid the dilution effect of cell passage, the transfected 293 cells were harvested at various time points without cell passage after the initial transfection. We extracted the replicating viral genome and to confirm its size, we used DpnI to remove non-replicating MCV DNA and EcoRI to linearize the full-length genome. The digested genomic DNA was then subjected to a Southern blot using a MCV probe for hybridization (**Fig 1B**). Both MCVΔsT (~5.0 kbp) and wild type MCV (MCV_WT_, ~5.4 kbp) initiated replication and increased the DNA genome by day 2, consistent with successful initiation of viral genome replication from the transfected re-circularized MCV. However, after day 2, MCVΔsT could not maintain its replication activity, and its viral genome copy number decreased over the 8-day experimental time course. By contrast, MCV_WT_ replicated further, increasing its genome copies in transfected 293 cells (**Fig 1B**). As expected, no replication activity was detected from MCV_rep-_, in which LT cannot bind to the replication origin, rendering it unable to initiate DNA replication. We detected a comparable amount of DpnI-sensitive template DNA on day 1 and day 2 between the three transfected MCV DNAs, confirming equal transfection efficiency. These results suggest a critical role of sT in maintaining the viral genome.

### sT deletion attenuates viral early and late mRNA expression, inhibiting capsid protein production

**Fig 2:**
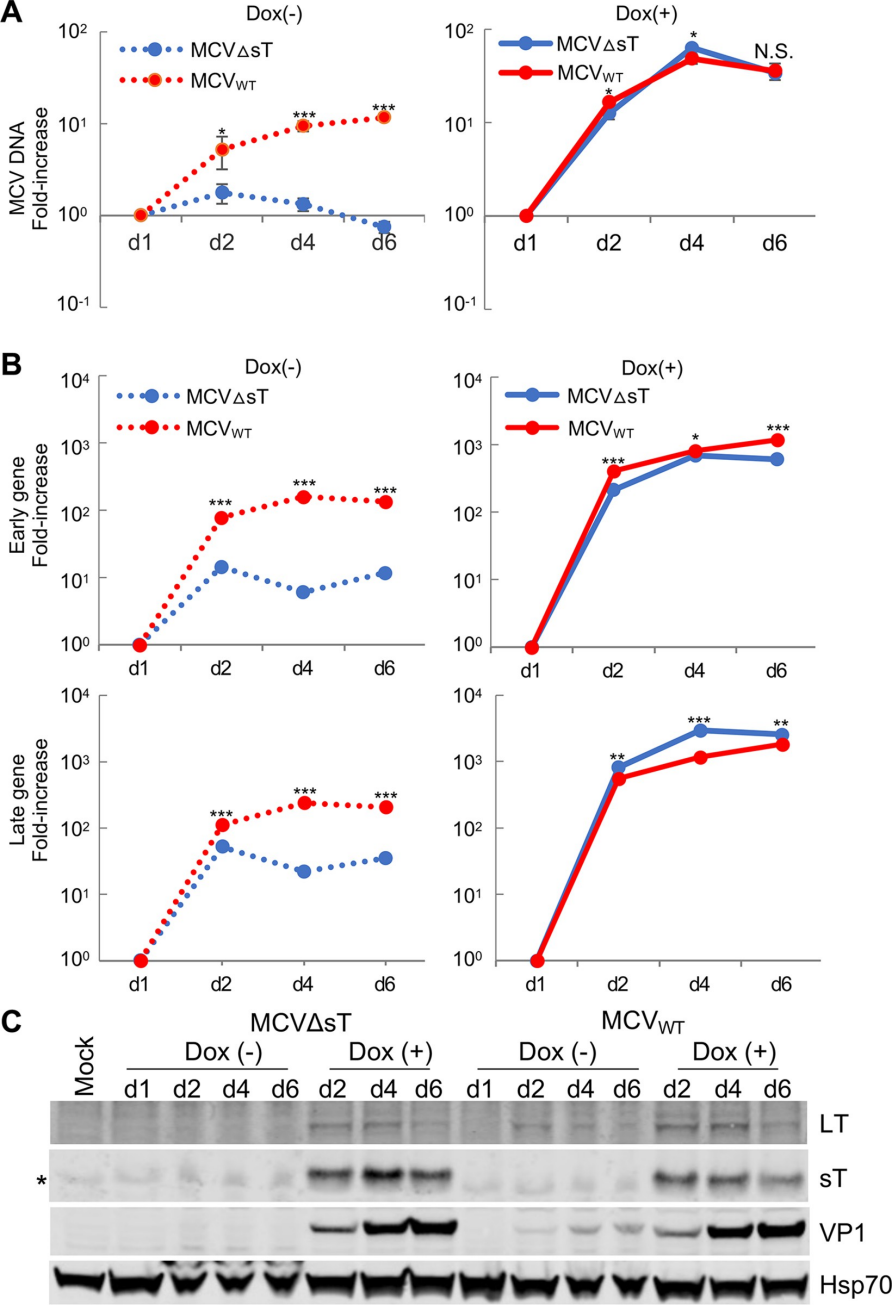
Exogenous sT expression rescues viral DNA replication and mRNA expression in MCVΔsT. MCV_WT_ and MCVΔsT viral DNAs were transfected into TRE-sT cells seeded for a 6-day time course experiment. Cells were harvested 24 h p.t. and the remaining cells were treated with or without doxycycline (Dox) at day 1 (0.5 μg/mL) and paired samples were harvested at day 2, day 4, and day 6 p.t.. Time kinetics in DNA replication, gene expression, and protein expression in both MCV_WT_ (red lines) and MCVΔsT (blue lines) were analyzed. Unpaired T-test was used for statistical analysis. *, P<0.05; **, P<0.01; ***, P<0.001. **(A)** Extracted genomic DNAs were digested with DpnI and EcoRI and subjected to qPCR using primer sets detecting MCV VP2 and GAPDH genes. Relative MCV DNA copy number to the day 1 sample was determined by the 2^-ΔΔCt^ method using GAPDH for normalization. **(B)** RNA was extracted and used to synthesize cDNA for qRT-PCR to determine early and late gene mRNA expression levels using MCV PanT and VP2 (set 1) primer pairs, respectively. Dotted lines represent Dox- condition while solid lines show Dox+ condition. Primers used in each experiment are listed in **S1 Table**. Relative MCV DNA copy number and early and late gene levels to the day 1 sample were determined by the 2^-ΔΔCt^ method using 18S RNA for normalization. **(C)** Protein was extracted and subjected to an immunoblot showing LT, sT, and VP1 expression levels. Immunoblots were stained with LT (CM2B4), sT (2T2) and VP1 (CM9B2) antibodies with Hsp70 antibody as a loading control. Asterisk (*) indicates non-specific band.

To examine if expressing the sT protein exogenously could rescue MCVΔsT replication, we established 293 TRE-sT cells that inducibly express codon-optimized sT by doxycycline treatment. Cells were seeded in seven dishes and transfected with the MCVΔsT and MCV_WT_ genomes. We harvested cells from one dish at day 1 post-transfection (p.t.) and added either doxycycline or autoclaved water to the remaining three dishes. To analyze viral growth kinetics relative to day 1, cells with or without sT expression were harvested at days 2, 4, and 6 p.t. for viral DNA, RNA, and protein expression analyses. In the absence of sT expression, MCVΔsT DNA increased moderately by day 2, yet decreased below the starting copy number by day 6 (**Fig 2A**, **left panel)**, consistent with the Southern blot assay **(Fig 1B)**. However, MCV_WT_ DNA steadily increased over the course of the 8-day experiment (**Fig 2A**, **left panel)**. Interestingly, induction of sT expression by doxycycline significantly increased both MCVΔsT and MCV_WT_ DNA, indicating that sT expression rescued MCVΔsT replication and further activated MCV_WT_ viral replication **(Fig 2A, right panel).**

Using RNA from the same samples, we also examined early and late gene expression using primers targeting the panT (sT and LT) and VP2 transcripts. Both early and late transcripts were significantly lower in MCVΔsT when compared to MCV_WT_ **(Fig 2B, left panels)**. It is possible that the decrease in early gene expression is due in part to the deletion of the LT intronic sequence that encodes sT [19]. Exogenous sT expression fully activated both early and late gene expression in MCVΔsT and MCV_WT_ (**Fig 2B, right panels)**. Between days 1 and 6, the magnitude of viral mRNA increase by sT (~1000-fold) was significantly higher than that of viral DNA increase (~10-fold). Immunoblots of these samples further revealed a lack of LT and VP1 protein expression in MCVΔsT **(Fig 2C)**. While LT and VP1 protein expression was detectable after day 2 p.t. in MCV_WT_ in the absence of sT induction, no protein expression was detected from MCVΔsT. Doxycycline induction of sT rescued LT and VP1 expression in MCVΔsT and further increased protein expression in MCV_WT_. These results confirm that sT deletion does not affect LT protein expression. The levels of LT and VP1 protein induced by sT at each time point are nearly identical between MCVΔsT and MCV_WT_, coordinating with the corresponding viral early and late gene expression that are fully activated by sT induction **(Fig 2B**). In the absence of doxycycline, no endogenous sT was detected **(Fig 2C)**. Although LT protein expression decreased at day 6, VP1 protein expression increased in sT-induced MCVΔsT, as well as sT-induced and sT-noninduced MCV_WT_ transfected cells, consistent with progression of the viral life cycle.

### The LTSD domain of MCV sT activates viral gene expression

**Fig 3:**
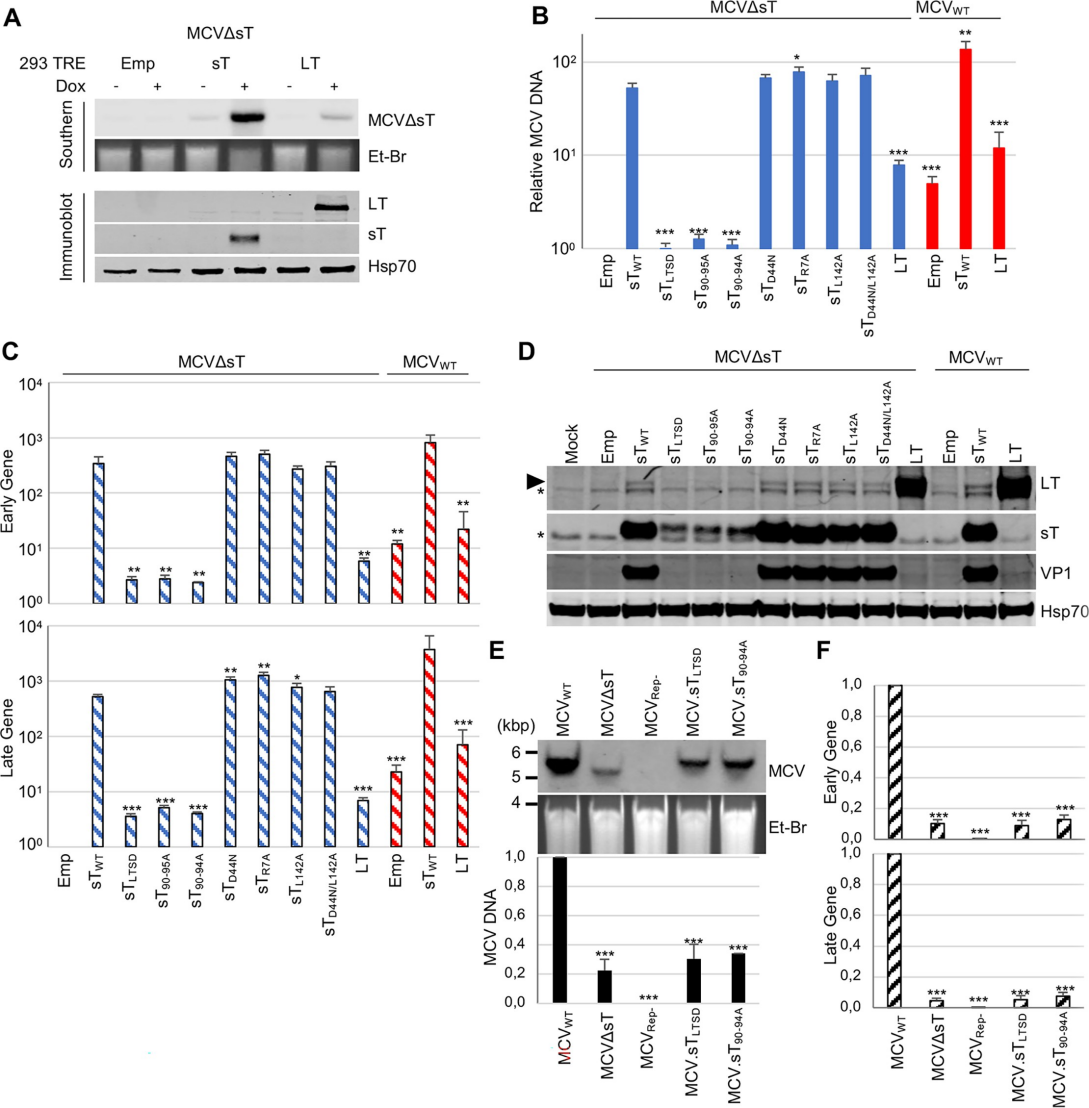
Mapping of sT domain required for viral genome maintenance. **(A)** MCVΔsT DNA was transfected into 293 TRE-Emp, sT, and LT cells and harvested at day 6 p.t.. gDNA was digested with EcoRI and DpnI and run on a Southern blot showing the effect of exogenous pcDNA sT and LT on MCVΔsT DNA replication. Immunoblot shown below was performed to confirm expression of exogenous sT and LT levels. **(B-D)** 293 cells were co-transfected with MCV_WT_ (red) or MCVΔsT (blue) and various pcDNA6 sT mutants including pcDNA Empty (Emp). Cells were then harvested at day 4 p.t.. **(B)** qPCR was performed as described in Fig 2A legend. To determine the effect of sT, sT mutants, and LT on MCVΔsT and MCV_WT_ replication, MCV DNA levels, relative to the control sample (MCVΔsT co-transfected with pcDNA Empty), were analyzed by 2^-ΔΔCt^ method. Solid bar graphs represent viral DNA. Unpaired T-test was used for statistical analysis. Significance was determined in comparison to MCVΔsT co-transfected with pcDNA6 sT_WT_. *, P<0.05; **, P<0.01; ***, P<0.001. **(C)** RNA was extracted and converted to cDNA for qRT-PCR analysis to determine early and late gene expression as described in Fig 2B legend. Relative early and late mRNA expression to the same control in Fig 3B was determined by the 2^-ΔΔCt^ method. Hatched bar graphs represent viral mRNA expression. **(D)** Corresponding protein levels shown on an immunoblot stained with MCV proteins (LT, sT, VP1) of each sample. Hsp70 was used as an internal control. A closed arrowhead indicates LT protein, and asterisk (*) designates nonspecific band. **(E)** 293 cells were transfected with MCT_WT_, MCVΔsT, MCV_rep-_, and two MCV mutants with different LTSD mutations (MCV.sT_LTSD_ and MCV.sT_90-94A_) and harvested day 4 p.t. for Southern blot using an MCV probe. The same samples were analyzed by qPCR to quantitate MCV DNA levels as in Fig 3B. Relative MCV DNA abundance to MCV_WT_ is shown. **(F)** With the same samples used in Fig 3E, total RNA was extracted and converted to cDNA for qRT-PCR analysis of early and late gene expression as in Fig 3C. Relative early and late gene expression to MCV_WT_ is shown. All error bars indicate standard error of three independent experiments.

To determine if LT stabilization is the only mechanism that activates viral replication by sT, we examined if the exogenous expression of LT could rescue MCVΔsT replication. We transfected MCVΔsT in 293 TRE-LT cells that inducibly express codon-optimized MCV LT by doxycycline as well as 293 TRE-sT cells and 293 TRE-Empty (Emp) control cells. Doxycycline was added at day 1 p.t., and samples were collected 4 days p.t. to examine viral replication by Southern blot. Immunoblots confirmed exogenous sT and LT expression induced by doxycycline treatment **(Fig 3A, bottom panel).** In the Southern blot, sT expression activated MCVΔsT replication substantially, indicating a full rescue by sT **(Fig 3A, top panel)**. LT did not activate MCVΔsT replication to the same extent. These results suggest that LT stabilization is not the sole mechanism by which sT activates viral DNA replication. MCV sT binds to various host factors such as PP2A, Fbw7, and the EP400/NuA4/Tip60 histone acetyltransferase (HAT) complex. The protein domains of sT that are responsible for host protein binding were mapped in previous studies [5,6,20]. We first examined if sT domain mutants could stabilize LT in 293 cells (**S1A Fig**). When codon-optimized sT and LT expression vectors were co-transfected in 293 cells, sT increased LT protein levels by stabilizing LT [11]. As shown in S1A Fig, the LT protein was stabilized by the codon-optimized sT mutants, which ablate PP2A (sT_R7A_, sT_L142A_), Hsp70/DnaJ (sT_D44N_), and both PP2A/Hsp70 (sT_L142A/D44N_) protein interactions. Using an original sT_LTSD_ mutant (sT_91-95A_), we confirmed the importance of the LTSD domain (aa 91–95), which interacts with Fbw7 [11], for LT stabilization. We also developed two additional LTSD mutants that cannot stabilize LT surrounding aa 90–95: sT_90-95A_ and sT_90-94A_ (**S1A Fig**). sT_90-95A_ has been reported as an EP400/NuA4/Tip60 interacting mutant along with two additional mutants sT_83-88A_ and sT_4M_ that have alanine substitution mutations at amino acid residues 83-88A and serine substitution mutations at residues 86, 87, 92, and 93 (4M) [20]. We found that sT_83-88A_ and sT_4M_ stabilized LT, whereas sT_90-95A_ did not **(S1A Fig)**.

To determine the sT domain that controls native viral replication, we performed phenotype rescue experiments by co-transfecting MCVΔsT with the aforementioned codon-optimized sT mutants. As shown in Fig 3B, in the empty vector co-transfected cells, replication of MCV_WT_ was nearly 5.0-fold greater than that of MCVΔsT, as determined by qPCR **(Fig 3B)**. When sT_WT_ was co-transfected, both MCVΔsT (50-fold) and MCV_WT_ (30-fold) DNA increased relative to their corresponding empty vector co-transfected cells. This indicates that exogenous sT expression in MCV_WT_, regardless of endogenous sT expression, can further increase viral DNA replication. When we co-transfected MCVΔsT with various domain mutants, only three LTSD-related mutants (sT_LTSD_, sT_90-95A_, and sT_90-94A_) failed to rescue MCVΔsT replication, whereas all DnaJ and PP2A binding mutants restored MCVΔsT replication to levels comparable to sT_WT_ **(Fig 3B)**. These results support the importance of the LTSD for MCV genome replication. Although co-expression of LT increased DNA replication for both MCVΔsT and MCV_WT_ genomes (7.9- and 12-fold), the levels were once again not comparable to those produced by co-expression of sT **(Fig 3B)**.

We also examined viral early and late gene expression by qRT-PCR **(Fig 3C)**. In the absence of exogenous sT and LT, early and late gene expression from MCV_WT_ was 12- and 23- fold higher, respectively, than in MCVΔsT. However, the difference in MCV DNA levels between MCVΔsT and MCV_WT_ was only 5.0-fold. This suggests that the decrease in early and late gene expression induced by sT deletion is not simply a reflection of viral template DNA reduction. Interestingly, rescue of exogenous LT expression increased both early and late gene expression 5.9- and 6.9-fold, respectively, and a corresponding 7.8-fold increase in MCV DNA levels was also seen. This supports the notion that LT activates both early and late gene expression by increasing viral DNA replication. In contrast to LT, sT co-expression markedly increased early and late gene expression 342- and 523-fold, respectively, indicating that sT activates both early and late gene expression by a post-viral DNA replication mechanism. Of the sT mutants, LTSD mutants (sT_LTSD_, sT_90-95A_, sT_90-94A_) failed to rescue early and late gene expression. All other DnaJ and PP2A mutants activated MCVΔsT mutants to a similar degree as sT_WT_ (**Fig 3C**). We also tested if SV40 sT (SV40 sT_WT_) or a PP2A binding mutant of SV40 sT (SV40 sT_L133A_), could rescue transcription in MCVΔsT. However, neither MCV early nor late gene expression could be rescued by SV40 sT (**S1B Fig**), confirming that MCV sT-mediated activation of viral transcription is independent of PP2A and specific to MCV sT. Therefore, host factors that promote viral replication downstream of sT are likely to be different between MCV and SV40. Immunoblots confirmed strong expression of exogenously expressed sT and LT proteins (**Fig 3D**). Endogenous expression of sT by MCV_WT_, however, was too low to be detected in transfected 293 cells. LT expression by MCV_WT_ was detected, yet it was significantly lower than exogenous LT. LT expression from MCVΔsT itself was lower than that from MCV_WT_ and was not detected (**Figs 2C and 3D**). Co-expression of sT constructs that rescued MCVΔsT replication (sT_WT_, sT_D44N_, sT_R7A_, sT_L142A_, and sT_D44N/L142A_) induced late VP1 protein expression significantly and increased LT expression minimally as compared to the pronounced LT stabilization seen in S1A Fig. By contrast, LTSD mutants failed to induce LT and VP1 expression (**Fig 3D**), consistent with attenuated early and late gene induction by the LTSD mutants (<3-fold for early gene and <5-fold for late gene) (**Fig 3C**). Although the LTSD appears to regulate viral gene expression and replication, expression of LTSD mutant protein levels were significantly lower than wild type sT and other mutants that rescued MCVΔsT transcription and replication (Fig 3D). Thus, we titrated the sT_WT_ and LTSD mutants’ expression vectors to determine the levels of sT that would rescue the MCVΔsT phenotype. MCVΔsT was co-transfected with different amounts of sT expression vectors to achieve comparable protein expression levels across sT_WT_ and different sT mutants in 293 cells (**S1C Fig**). Three LTSD mutants, sT_LTSD_, sT_90-95A_, and sT_90-94A_ required 2 ~ 20-fold sT_WT_ expression vectors for transfection in order to express proteins comparable to sT_WT_ (**S1C Fig**). Strikingly, a small amount of sT_WT_ protein (10 ng of vector co-transfection) was sufficient to rescue LT and VP1 protein expression, whereas higher sT_LTSD,_ sT_90-94A,_ and sT_90-95A_ protein expression (500 ng of expression vector co-transfection) did not rescue LT and VP1 protein expression. We also examined if sT_WT_ restoration of LT and VP1 protein expression accompanies increased viral gene expression. In line with the protein expression results, a small amount of sT expression fully activated early and late gene expression, while the LTSD mutants could not activate early and late gene expression, regardless of protein levels (**S1B Fig)**. Although endogenous sT protein expression from MCV_WT_ was undetectable by immunoblot (**Figs 2C and 3D**), these results suggest that even a small amount of sT_WT_ can sufficiently activate MCV gene expression and replication, and that the LTSD is required to stimulate viral DNA replication, gene expression, and protein expression.

Since the LTSD may rescue MCVΔsT replication, we examined if the LTSD is the sole domain controlling sT’s genome maintenance function. To investigate this, we introduced two LTSD mutations into the MCV full genome to generate MCV.sT_LTSD_ and MCV.sT_90-94A_, and compared their replication to that of MCVΔsT in transfected 293 cells (**Fig 3E**). In a Southern blot, replication of the two LTSD mutants, as compared to MCV_WT_, was significantly reduced to levels comparable to MCVΔsT (**Fig 3E, top panel**). qPCR analysis over three replicates of samples also confirmed a similar reduction (70~80%) in viral DNA replication for LTSD mutants and MCVΔsT (**Fig 3E, bottom panel**). When we examined early and late gene expression with the same samples, early gene expression from the LTSD mutants and MCVΔsT was reduced by ~90% while late gene expression was reduced by ~95% (**Fig 3F**). A larger reduction in viral gene expression by sT deletion and LTSD mutations suggests that viral gene expression might be sT’s primary regulatory target for MCV genome maintenance.

### LT binding to the NCCR is a prerequisite for sT-mediated activation of viral gene expression

**Fig 4:**
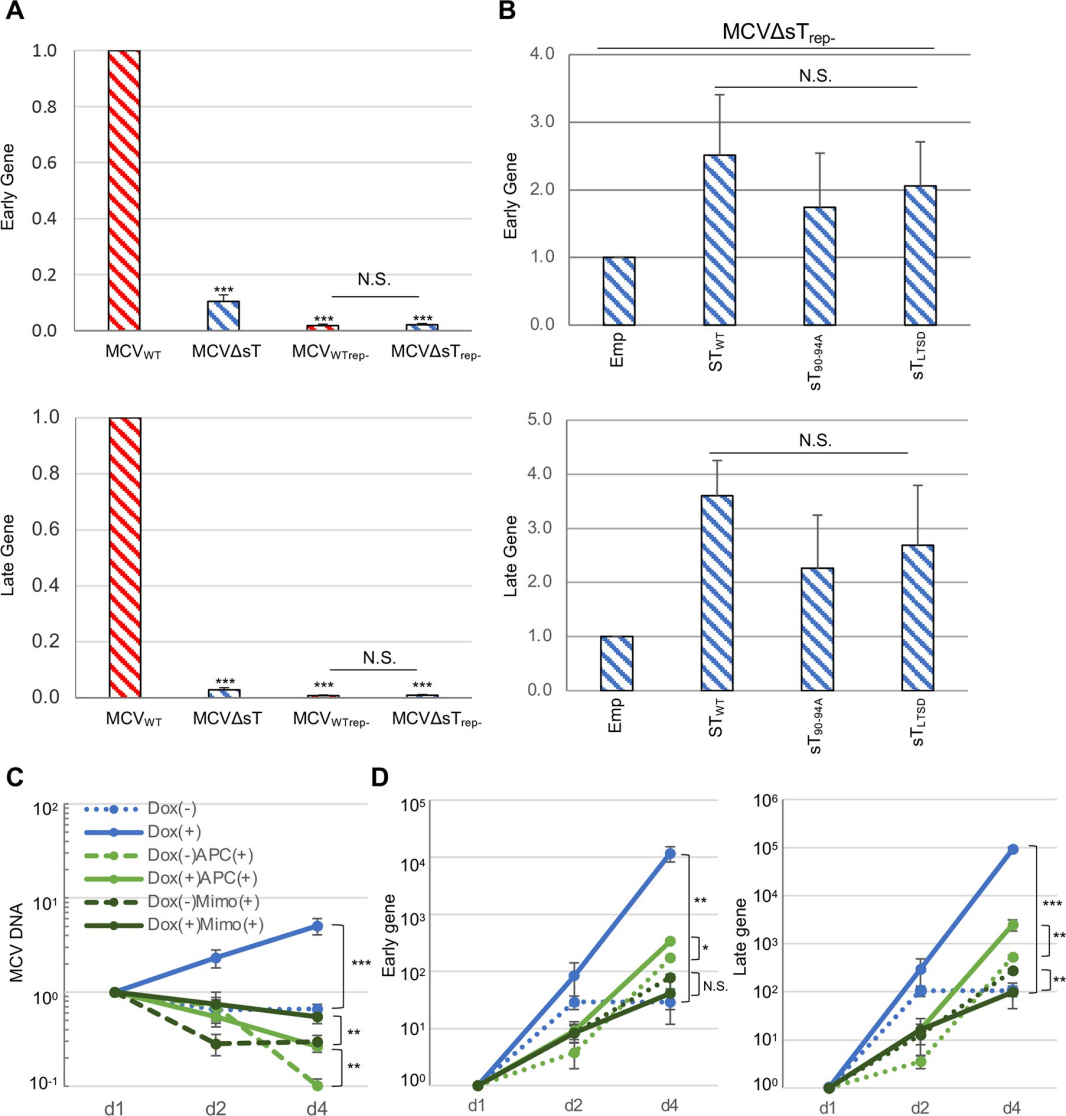
sT requires viral DNA replication for an efficient activation of viral gene expression. **(A)** 293 cells transfected with MCV_WT_, MCVΔsT, and their rep- counterparts were harvested at day 4. Total extracted RNA was subjected to qRT-PCR analysis by the 2^-ΔΔCt^ method with 18S ribosomal RNA for normalization. Relative mRNA expression to the MCV_WT_-transfected sample is shown. Error bars indicate SD. **(B)** 293 cells co-transfected with MCVΔsT_rep-_ and either pcDNA empty (Emp), pcDNA sT_WT_, or LTSD mutants (pcDNA sT_90-94A_ and pcDNA sT_LTSD_) were harvested at day 4. Early and late gene expression was determined as in Fig 4A. Relative mRNA expression to the pcDNA6 empty co-transfected sample is shown. **(C)** 293 TRE-sT cells that can inducibly express sT by doxycycline were transfected with MCVΔsT in 13 dishes. At day 1 p.t., cells in one dish were harvested and doxycycline (0.5 μg/mL), DNA inhibitors (5 μM aphidicolin (APC) and 400 μM mimosine (Mimo)), and water (for mock) were added to the other 12 dishes. The remaining cells were harvested at day 2 and 4 p.t. To examine viral DNA replication, qPCR was performed, as described in the Fig 2A legend. **(D)** Using the same samples as in Fig 4C, RNA was extracted and converted to cDNA for qRT-PCR analysis, as described in the Fig 2B legend, to determine early and late gene expression.

To determine how LT function relates to sT function, viral gene expression in two replication-defective mutants, MCV_WTrep-_ and MCVΔsT_rep-_, was compared by qRT-PCR to that in MCV_WT_ and MCVΔsT in transfected 293 cells. As seen in Fig 3F, sT deletion reduced early and late gene expression by ~90% and ~97%, respectively. Both rep- mutants showed an even greater decrease with no discernible difference between MCV_WTrep-_ and MCVΔsT_rep-_, indicating that the effect of sT was cancelled (**Fig 4A**). We also examined if sT could rescue viral gene transcription in MCVΔsT_rep-_ in the absence of LT binding to the viral genome. qRT-PCR demonstrated that although sT co-expression activates early and late gene expression over 100-fold in MCVΔsT (**Fig 3C**) (~341-fold for early and ~522-fold for late gene expression), a single rep- mutation introduced into the MCVΔsT genome drastically reduced sT’s viral gene activation ability to only a few-fold **(Fig 4B) (**~2.5-fold for early and ~3.5-fold for late gene expression). While subtle activation was seen in early and late gene expression by sT_WT_ co-expression (**Fig 4B**), two LTSD mutants, an original sT_LTSD_ and sT_90-94A_, also increased viral gene expression moderately. These results indicate that LT binding to the NCCR is essential for sT to activate viral gene expression.

We further confirmed this result by using a reporter MCV that expresses a fluorescent protein under a late promoter, since sT is essential for VP1 protein production. We generated both wild type and ΔsT MCV that encode the ZsGreen (ZsG) fluorescence reporter protein-FMDV 2A(F2A) sequence under the late gene promoter before the VP2 gene (**S2A Fig)**, MCV_WT_.L-ZsG and MCVΔsT.L-ZsG). Although ZsG-F2A insertion ablated VP1 protein expression, normal viral replication occurred from the MCV.L-ZsG genome [18]. When transfected into 293 cells, MCVΔsT.L-ZsG showed remarkably lower fluorescence than MCV_WT_.L-ZsG, consistent with the significant reduction of late gene expression seen in the native MCV genome (**S2A Fig**). While ZsG positivity was comparable between MCV_WT_.L-ZsG and MCVΔsT.L-ZsG, introduction of the rep- mutation in the two viral genomes (MCV_WT_.L-ZsG_rep-_ and MCVΔsT.L-ZsG_rep-)_ decreased ZsG positivity, ablating the sT effect as seen in the native MCV genome (**S2B Fig**). By contrast, the mean fluorescence intensity of ZsG was decreased by both sT deletion and rep- mutation (**S2C Fig**). MCV.L-ZsG is a useful reporter virus to assess late gene promoter activity, and the results in native MCV and MCV.L-ZsG reporter viruses confirm that LT binding to the viral origin of the NCCR is a prerequisite for sT function.

Next, using general DNA replication inhibitors, we examined if ongoing viral DNA replication, which follows LT binding to the NCCR, is important for sT-induced viral gene expression. MCVΔsT-transfected 293 TRE-sT cells in one of 13 dishes were harvested at day 1 p.t., and the others were treated with or without DNA replication inhibitors aphidicolin and mimosine in the presence or absence of sT induction with doxycycline to be harvested at days 2 and 4 p.t.. Treatment with aphidicolin and mimosine prevented sT-induced viral DNA replication and decreased viral DNA on days 2 and 4 relative to day 1 (**Fig 4C**). In contrast to this decrease in viral template DNA, early and late mRNA expression continued to increase over time, indicating that, in the absence of sT, viral mRNA expression is independent of viral template DNA. However, sT-mediated increase of viral mRNA expression was significantly attenuated by aphidicolin and mimosine (**Fig 4D**). In the polyomavirus late gene expression, wraparound transcription that runs through the polyA site and circles the genome multiple times are reported [21–23]. Thus, both positive and negative strand transcripts are expressed from the early gene locus. Since qRT-PCR exploits random hexamers for cDNA synthesis, we used a positive strand-specific primer for cDNA synthesis to detect positive strand early mRNA. Semi-quantitative RT-PCR using the aphidicolin and mock-treated RNA harvested at day 4 p.t. demonstrated that sT induction by doxycycline increases early T antigen mRNA, while aphidicolin ablates the increase without decreasing basal mRNA expression. No signal was seen in the negative control samples that depleted RT in the reaction, confirming that the PCR signals originated from viral mRNA as opposed to transfected template DNA (**S2C Fig**). These results suggest that sT-mediated viral gene activation requires concurrent viral DNA replication.

### MCVΔsT virions cannot maintain replication or induce VP1 protein expression in infected dermal fibroblast cells

**Fig 5:**
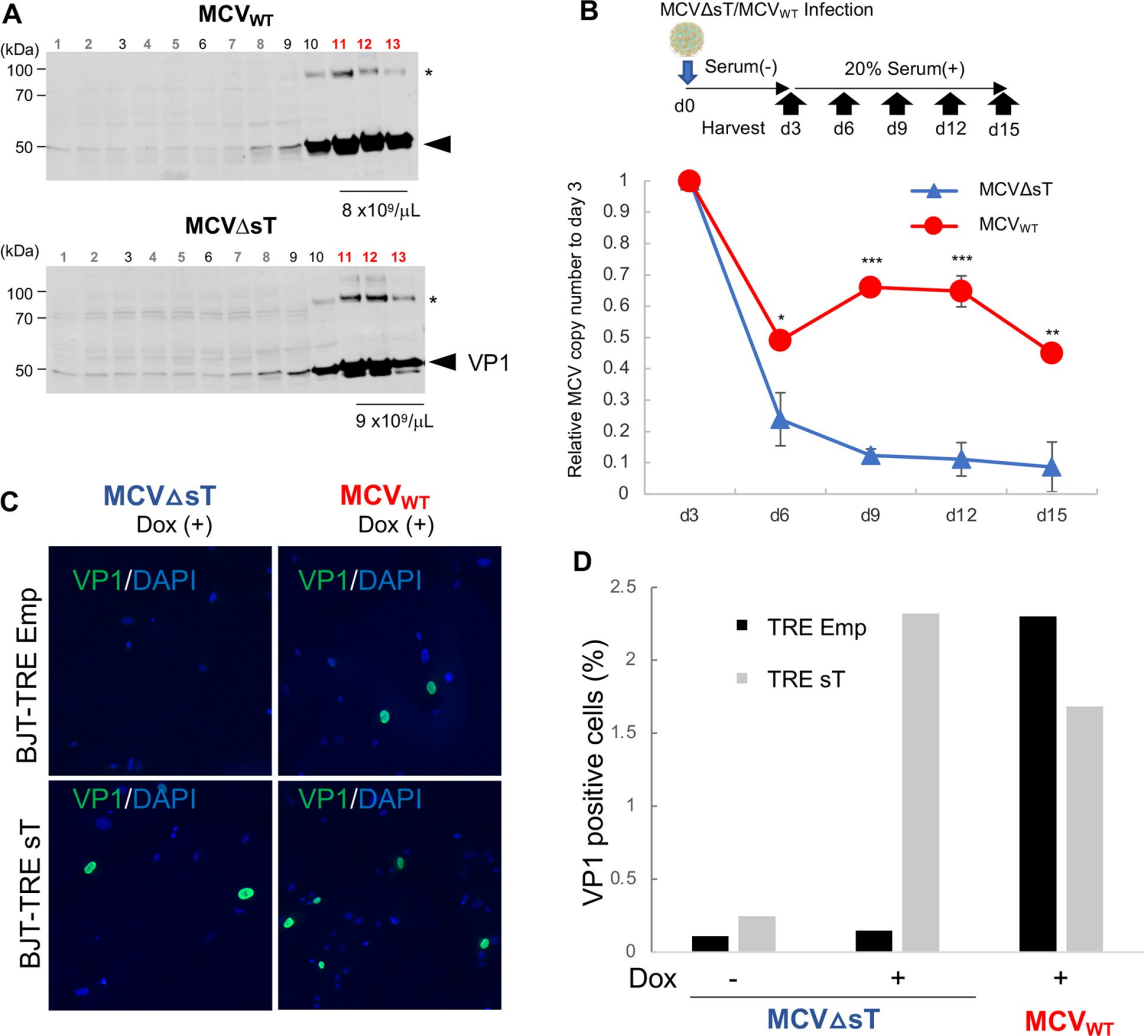
sT regulates VP1 expression in infected permissive cells. **(A)** Production of MCVΔsT and MCV_WT_ virions in transfected 293 TRE-sT cells with sT protein induction. Thirteen fractions from MCVΔsT- and MCV_WT_-transfected 293 TRE-sT cell lysates sedimented by Opti-prep (1~13 from lighter to heavier) demonstrate similar virion packaging efficiency in fractions 11~13 between MCV_WT_ and MCVΔsT according to VP1 protein expression and genome copy numbers from the pooled fractions (fractions 11~13). VP1 protein was detected by CM9B2 by immunoblot, and absolute MCV genome copy number was determined by qPCR with a VP2 primer (set 1) pair. *VP1 dimer protein. **(B)** BJ.hTERT cells were infected with 10^6^ per cell genome copies of MCVΔsT and MCV_WT_ and harvested over a time period of 15 days after the addition of serum on day 3 post infection (top panel). qPCR was performed on genomic DNA using the 2^-ΔΔCt^ method with GAPDH for normalization. A relative MCV DNA abundance to the day 3 post-MCV-infected sample indicates the inability of MCVΔsT to maintain the viral genome. Significance was determined by unpaired T test in comparison between MCVΔsT and MCV_WT_ within the same date point *, P<0.05; **, P<0.01; ***, P<0.001. **(C)** Exogenous sT rescues VP1 protein expression in MCVΔsT infected cells. BJ.hTERT TRE-Emp and TRE-sT cells infected with MCVΔsT and MCV_WT_ were treated with doxycycline at day 5 p.t. to induce exogenous sT expression. Cells were harvested at day 8 for immunofluorescence with VP1 antibody. **(D)** Doxycycline (Dox) rescues VP1 expression in the MCVΔsT mutant to levels greater than that of MCV_WT_. VP1 positive cells were counted to determine % positivity.

Dermal fibroblasts have been shown to be target cells for MCV infection [24]. We examined if sT is also important for MCV genome maintenance and persistence in dermal fibroblast cells that are permissive to MCV infection. In order to produce infectious MCV, MCVΔsT and MCV_WT_ viral genomes were transfected in 293 TRE-sT cells and cultured for 10 days under doxycycline-induced sT expression [17]. In the final step of virion purification by iodixanol gradient ultracentrifugation, similar amounts of MCVΔsT and MCV_WT_ virions were sedimented in fractions #11–13 as demonstrated by MCV VP1 immunoblots (**Fig 5A**). These data suggest that, in the presence of sT expression, MCVΔsT virion production is as efficient as MCV_WT_ virion production. After the three MCV-positive fractions were combined, a viral genome titer (MCV genome copies/μL) was determined by qPCR for subsequent infection studies.

For the infection studies, we used human foreskin BJ fibroblasts immortalized by human telomerase (BJ.hTERT) to avoid the effect of replicative senescence on MCV replication. As reported by Liu et al, 10^6^ copies of MCV_WT_ and MCVΔsT per cell were infected into BJ.hTERT cells with serum-free DMEM/F12 medium containing the GSK3 inhibitor CHIR99021 and 1 mg/mL collagenase IV for 3 days [17]. Cells were harvested every 3 days up to 15 days post-infection to examine MCV replication. To study MCV replication kinetics, the relative copy number of MCV at day 3 was determined by qPCR. It should be noted that even though MCVΔsT and MCV_WT_ were infected with the same copy number of MCV, the MCVΔsT DNA copy number was 1.5-fold higher than that of MCV_WT_ at day 3. The copy number of MCV_WT_ significantly decreased by 50% between days 3 and 5, indicating that only part of the viral genome initiates replication in infected cells. Despite this initial drop in the genome copy number, the MCV_WT_ genome was sustained between days 6 and 15 post-infection (**Fig 5B**). By contrast, the MCVΔsT genome copy number decreased by 80% between days 3 and 6 and continued to decrease until day 15. This indicates that the MCVΔsT genome cannot persist in infected cells. We also examined early and late gene expression in the MCV infected BJ.hTERT cells. In MCV_WT_-infected cells, both early and late gene expression was maintained until day 9 post-infection but decreased at day 12. In MCVΔsT cells, however, early and late gene expression was reduced by >95% at day 6 post-infection and continued to decrease over the course of the experiment (**S3A Fig**).

We also examined if exogenous sT induction could rescue VP1 capsid protein expression in MCVΔsT-infected fibroblast cells by establishing BJ.hTERT TRE-sT cells that inducibly express codon-optimized sT by doxycycline (**S3B Fig**). BJ.hTERT TRE-sT cells infected with MCVΔsT or MCV_WT_ were split into two wells and treated with or without doxycycline at day 5 post-infection. Cells were harvested at day 8 and examined for VP1 protein expression by immunofluorescence. In the absence of doxycycline, VP1 positivity was very low (<0.2%) in MCVΔsT-infected BJ.hTERT TRE-sT cells (**Fig 5C and 5D**). Doxycycline treated TRE-sT cells showed >10-fold VP1 positivity (2.2%), indicating that sT expression induces VP1 protein expression in infected BJ.hTERT cells. However, in the control MCVΔsT-infected BJ.hTERT TRE-Emp cells, VP1 positivity remained low irrespective of doxycycline treatment. Regardless of the cell lines infected, MCV_WT_ showed nearly 2% VP1 positivity in the presence of doxycycline (**Fig 5C and 5D**). These data suggest that, even in infected fibroblast cells, sT expression is required for VP1 capsid protein expression.

### Proximity biotin-labeling with sT_WT_ and sT_LTSD_

**Fig 6:**
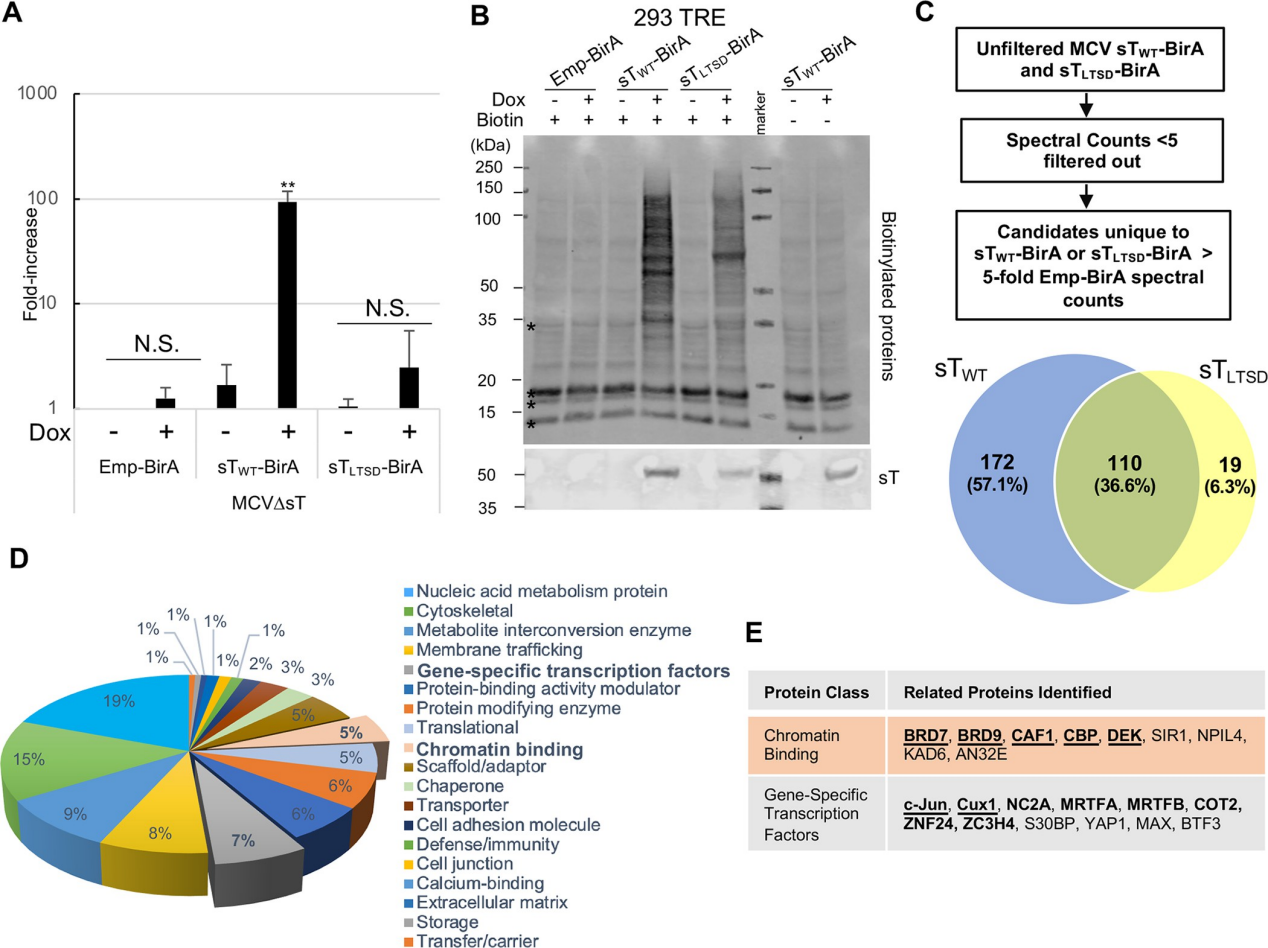
Identification of LTSD-dependent MCV sT proxisome by Bio-ID. **(A)** sT fusion with biotin-ligase BirA does not alter the function of sT. Prior to Bio-ID analysis, 293 TRE-sT_WT_-BirA, 293 TRE-sT_LTSD_-BirA, and 293 TRE-Emp-BirA cells were transfected with MCVΔsT, induced by doxycycline and harvested at day 4 p.t. qPCR with VP2 and GAPDH primers confirmed that MCV replication is still activated by sT_WT_-BirA, but not by TRE sT_LTSD_-BirA. Error bars indicate SD. **(B)** Host protein biotinylation is induced by sT-BirA fusion proteins only in the presence of biotin. Host cell biotinylation (top panel) and expression of sT-BirA fusion proteins (bottom panel). sT protein expression was confirmed by streptavidin and sT (2T2) immunoblots in sT_WT_-BirA, sT_LTSD_-BirA, and Emp-BirA cells after doxycycline and biotin treatment. **(C)** The flow chart of Bio-ID data analysis and Venn diagram summary of the distribution of related proteins identified by Bio-ID. 301 total proteins were identified as candidates for the sT_WT_ and sT_LTSD_ proxisome by the Bio-ID study. 57.1% were unique to sT_WT_, 6.3% were unique to sT_LTSD_, and 36.6% were common to both sT_WT_ and sT_LTSD_. **(D)** Gene ontology analysis for the sT proxisome identified by Bio-ID. The 172 unique to wild type sT interactors and the 110 interactors common to both wild type and the LTSD mutant as identified by the Bio-ID approach were included in the analysis. Proteins were analyzed using the PANTHER gene ontology software. Interactors were separated into 19 protein classes. **(E)** Summary of sT interactors identified as chromatin binding and transcription regulator proteins by the PANTHER software to use for further confirmational studies. Bolded proteins indicate proteins unique to sT_WT_. Underlined proteins were selected for further confirmational studies.

To identify the LTSD-dependent host protein interactions of MCV sT, we exploited a proximity-dependent biotin labeling assay (Bio-ID) [25] by fusing biotin-ligase BirA to the C-terminus of the sT protein. Prior to conducting the Bio-ID study, we confirmed that the C-terminal fusion of biotin-ligase BirA to sT did not alter the ability of sT to rescue MCVΔsT replication. First, we generated doxycycline-inducible 293 stable cells transduced with TRE-sT_WT_-BirA, TRE-sT_LTSD_-BirA, and TRE-Emp-BirA. MCVΔsT was transfected into the stable cell lines and replicated in the presence or absence of doxycycline. qPCR revealed increased MCV DNA by doxycycline induction of sT_WT_-BirA, but not by sT_LTSD_-BirA, indicating that BirA fused sT also activates viral replication through an LTSD-dependent mechanism (**Fig 6A**). BirA fusion, then, does not alter the function of the sT domain. Immunoblots also confirmed host protein biotinylation induced by sT_WT_-BirA and sT_LTSD_-BirA in doxycycline-treated 293 TRE stable cells in the presence of both doxycycline and biotin (**Fig 6B**). After purifying the biotinylated protein with streptavidin beads, we observed similar protein banding patterns between sT_WT_-BirA and sT_LTSD_-BirA, yet some bands were more intensive or specific to sT_WT_-BirA (**S4A Fig**, **asterisks**). These data suggest that mutating the LTSD may alter the proximity-dependent host protein biotinylation sT-BirA.

For the Bio-ID study, purified proteins were subjected to LC-MS/MS to identify potential host proteins that localize near sT_WT_ and sT_LTSD_. Proteins identified from the Empty-BirA control were used as a background.

Based on the criteria outlined in Fig 6C, out of a total of 3,239 proteins, 301 proteins were identified as potential sT interactors. 172 (57.1%) proteins were deemed to be specific to sT_WT_-BirA, while 19 (6.3%) were specific to sT_LTSD_-BirA. Among them, 110 (36.6%) proteins were found in common between sT_WT_-BirA and sT_LTSD_-BirA. While the majority of sT_LTSD_-BirA specific proteins overlapped with sT_WT_-BirA (110/129, 85.3%), less than half of the sT_WT_-BirA proteins (110/282, 39.0%) were shared with sT_LTSD_-BirA, indicating that more proteins were uniquely labeled by wild type sT_WT_-BirA in an LTSD-dependent mechanism. Ablating the LTSD domain showed a decrease in the number of protein interactors able to associate with sT. We also identified known MCV sT interactors including PP2A, EP400, Max, and PP4C. However, since PP2A and PP4C proteins were also identified from control samples, they were excluded from the analysis even though greater spectral counts were seen in sT_WT_ than in the control samples (S1 **Dataset**).

Next, to explore the cellular functions potentially affected by sT, we performed a Gene Ontology (GO) analysis using the PANTHER software to classify the 301 proteins that were found to have a greater than 5-fold difference in spectral counts between sT and Empty from the Bio-ID study. PANTHER revealed multiple protein classes that were potential interactors of sT **(Fig 6D)**. The top 10 protein classes identified were: nucleic acid metabolism (19.3%), cytoskeleton (14.6%), metabolite interconversion (9.4%), membrane trafficking (8.2%), transcriptional regulation (7.0%), protein-binding activity modulation (6.4%), protein modifying enzyme (6.4%), chromatin binding (5.3%), scaffold/adaptor (5.3%), and translation (5.3%). Within major protein classes, we identified subclasses to determine if specific protein functional groups may be important for the sT phenotype **(S4B Fig).** Since the four largest protein classes identified by PANTHER comprised greater than 50% of the total proteins identified, we further studied the subclassifications for these groups. For these major protein classes, sub-classification group names are listed with corresponding percentages of total proteins per class to discuss potential cellular functions affected by sT **(S4B Fig)**. Since our results suggest that sT activates early and late viral mRNA expression, sT may interact with gene expression regulators including gene specific transcription factors and chromatin binding proteins **(Fig 6E)**. To verify that there were no false positives among the potential interactors, the spectral count of each was compared to the average spectral counts listed in the CRAPome 2.0 using the REPRINT (resource for evaluation of protein interaction networks) website **(S4C Fig)**. All of the identified transcriptional regulators and chromatin-binding factors demonstrated greater spectral counts than previously reported CRAPome averages, except for MRTFA and MRTFB.

### LTSD-dependent nuclear sT interaction with chromatin and transcriptional regulators in MCV replication

From these potential protein classes, we selected chromatin binding proteins and transcriptional regulators to validate the proximity-ligation assay results (**Fig 6E**). Of the factors unique to sT_WT_ **(Fig 6E, bolded)**, we investigated transcriptional regulators Cux1 and c-Jun, and chromatin binding proteins BRD7, BRD9, CHAF1A, CBP and DEK (**Fig 6E, underscored**) by immunoprecipitation assays to study their physical binding. Despite CBP’s similarity to EP300, CBP-specific peptides were only detected in sT_WT_-BirA by mass spectrometry. EP300-specific peptides were found in both sT_WT_-BirA and the Empty-BirA background, and therefore excluded from our Bio-ID analysis according to our cut-off criteria.

**Fig 7:**
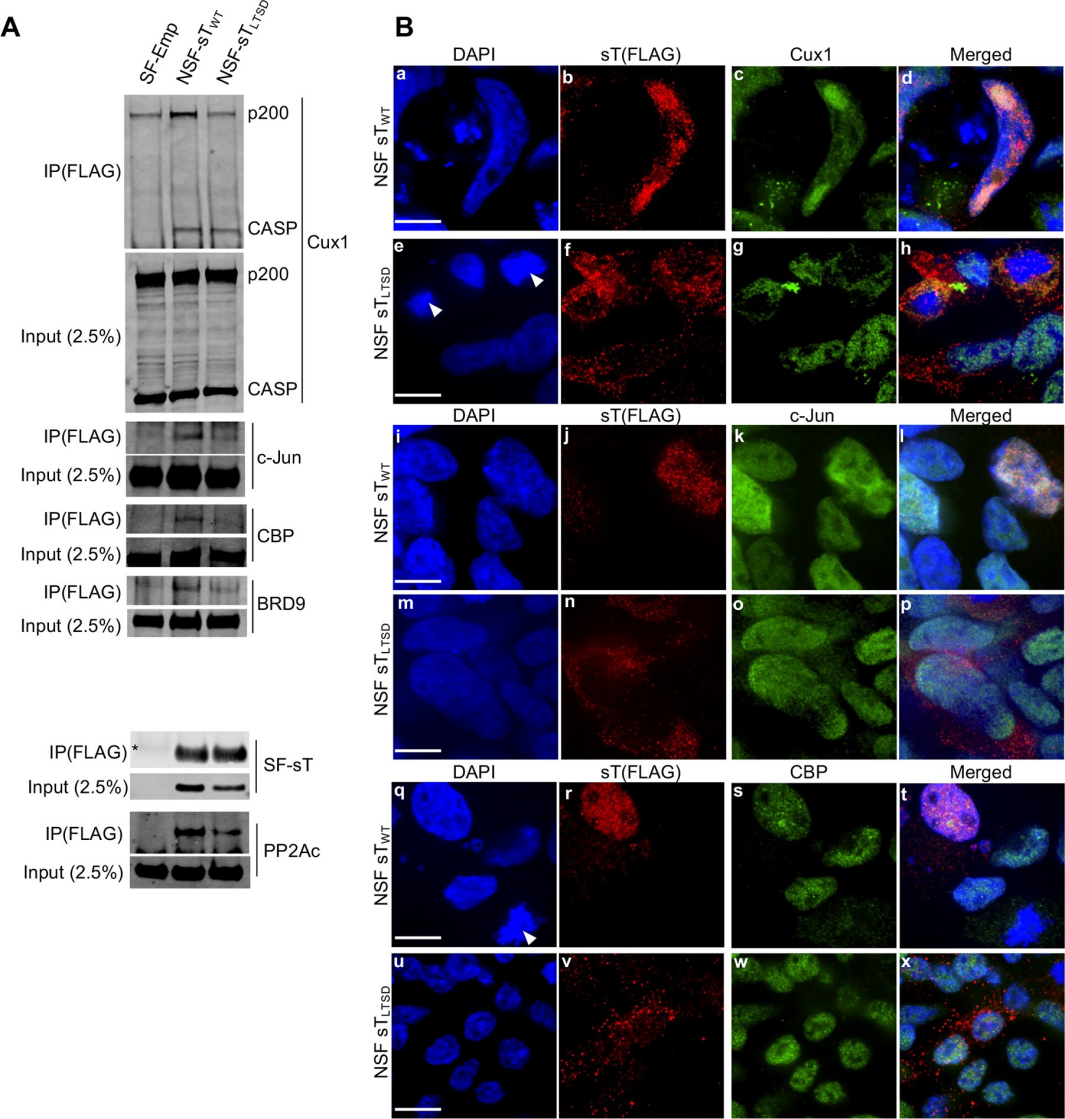
Validation of sT protein interaction with chromatin binding and gene-specific transcription factor proteins identified by Bio-ID. **(A)** Confirmation of LTSD-dependent direct sT_WT_ interaction with Cux1, c-Jun, CBP, and BRD9 by immunoprecipitation (IP). N-terminally SF-tagged (NSF) NSF-sT_WT_ and NSF-sT_LTSD_ cell lysates were immunoprecipitated with FLAG Ab. 2.5% of IP lysate was used as input. SF-sT was pulled down to confirm equal precipitation of sT_WT_ and sT_LTSD_. PP2Ac, which interacts with both sT_WT_ and sT_LTSD_, was used as a positive control. **(B)** MCVΔsT transfected 293 TRE NSF-sT_WT_ and NSF-sT_LTSD_ cells were treated with Dox at day 1 p.t. and harvested day 4 p.t. for immunofluorescence. Cells were co-stained with antibodies against FLAG sT (red, Alexa 488) and either Cux1, c-Jun, or CBP (green, Alexa 568), and images were captured by confocal microscopy. Magnification 100x. Arrowheads designate sT-positive mitotic cells. White bars on DAPI images indicate 10 μm.

We used doxycycline-inducible 293 stable cell lines that express StrepII-FLAG-tagged empty (SF-empty), C- and N-terminally SF-tagged codon-optimized sT_WT_ (CSF-sT_WT_ and NSF-sT_WT_), and N-terminally tagged sT_LTSD_ (NSF-sT_LTSD_) to perform immunoprecipitation (IP). CSF-sT_LTSD_ was not used because its protein expression was remarkably lower than that of NSF-sT_LTSD_. We preliminarily tested the potential interactors using CSF- and NSF-sT_WT_ (**S5A Fig)**, and if an interaction was detected, we used NSF-sT_WT_ and NSF-sT_LTSD_ to confirm LTSD-dependent interaction. The FLAG antibody, which immunoprecipitated similar amounts of NSF-sT_WT_ and NSF-sT_LTSD_, as well as sT’s known interactor, PP2Ac [11] (**Fig 7A, bottom panel),** co-precipitated Cux1, c-Jun, CBP, and BRD9 proteins robustly with NSF-sT_WT,_ but faintly with NSF-sT_LTSD._ These results suggest an LTSD-dependent interaction between sT and these four proteins. Cux1 is unique because it possesses multiple isoforms through proteolytic cleavage and differential splicing [26,27]. The Cux1 polyclonal antibody that recognizes the N-terminus of Cux1 protein detected full-length 200 kDa (p200-Cux1) and previously defined CASP (CDP/cut alternatively spliced product, 70kDa) isoform, an alternatively spliced product of Cux1 that localized at the Golgi apparatus [28]. Despite the background interaction with SF-empty, the p200-Cux1 repeatedly demonstrated a stronger interaction with NSF-sT_WT_ when compared to NSF-sT_LTSD,_ similar to other interactors. However, for CASP, a similar level of protein was co-precipitated with NSF-sT_WT_ and NSF-sT_LTSD_. PP2Ac was used as a positive control as it has been shown to interact with MCV sT [6].

Since NSF-sT_LTSD_ was weakly co-precipitated with potential interactors, we sought to further confirm sT’s LTSD-dependent interaction with Cux1, c-Jun, and CBP by immunofluorescence (IF). BRD9 was excluded from this experiment since no IF antibodies are available. To study their subcellular localization, we co-stained MCV sT and MCV LT with Cux1, c-Jun, and CBP in MCVΔsT-transfected 293 TRE-NSF-sT cells harvested at day 4 p.t., and Z-stack scanning was performed by confocal microscopy. As shown in previous reports (5, 24), NSF-sT_WT_ predominantly localized in the nucleus **(Fig 7Bd, 7Bl, and 7Bt)**. By contrast, NSF-sT_LTSD_ consistently showed more predominant cytosolic staining (**Fig 7Bh, 7Bp, and 7Bx)**. These results suggest that the LTSD domain potentially regulates nuclear sT protein expression by affecting nuclear protein stability or nuclear import. All three transcriptional regulators predominantly localized in the nucleus and showed a partial nuclear co-localization with NSF-sT_WT_ (**Fig 7Bd, 7BI, and 7Bt**), showing strong Pearson’s correlation coefficient (r >0.5) **(S5B Fig)**. Due to low nuclear expression, NSF-sT_LTSD_ protein showed no co-localization with the c-Jun or CBP, but Cux1 protein co-localization was seen in the cytosol of mitotic cells (**Fig 7Bh)**. This phenotype was not observed with c-Jun or CBP (**Fig 7Bp and 7Bx),** consistent with the IP results in which CASP was the only interactor to both sT_WT_ and sT_LTSD_ **(Fig 7A)**. CBP and c-Jun localized in the cytosol in mitotic cells, but no noticeable co-localization with NSF-sT_LTSD_ was observed in these areas **(Fig 7Bm-p and 7Bu-x)**. Using LT staining, we also examined if these interactors co-localize with replication foci in the nucleus (25). Replication foci were detected in the MCVΔsT-transfected 293 NSF-sT_WT_ cells, but not in the MCVΔsT-transfected NSF-sT_LTSD_ cells except for a diffuse nuclear signal (**S5C Fig**). All three proteins co-localized with LT replication foci. Curiously, Cux1 and c-Jun protein showed distinctive accumulation at the LT replication foci in addition to diffused nuclear expression (**S5C Fig**), suggesting an accumulation of Cux1 and c-Jun at the site of viral DNA replication and transcription. Both the IP and IF analyses validated our Bio-ID study, showing that four out of the seven candidate proteins analyzed interact with sT in the nucleus, and that LTSD mutation suppresses these interactions by reducing sT protein expression in the nucleus.

### Role of sT-interacting transcription regulators in MCV gene expression

**Fig 8:**
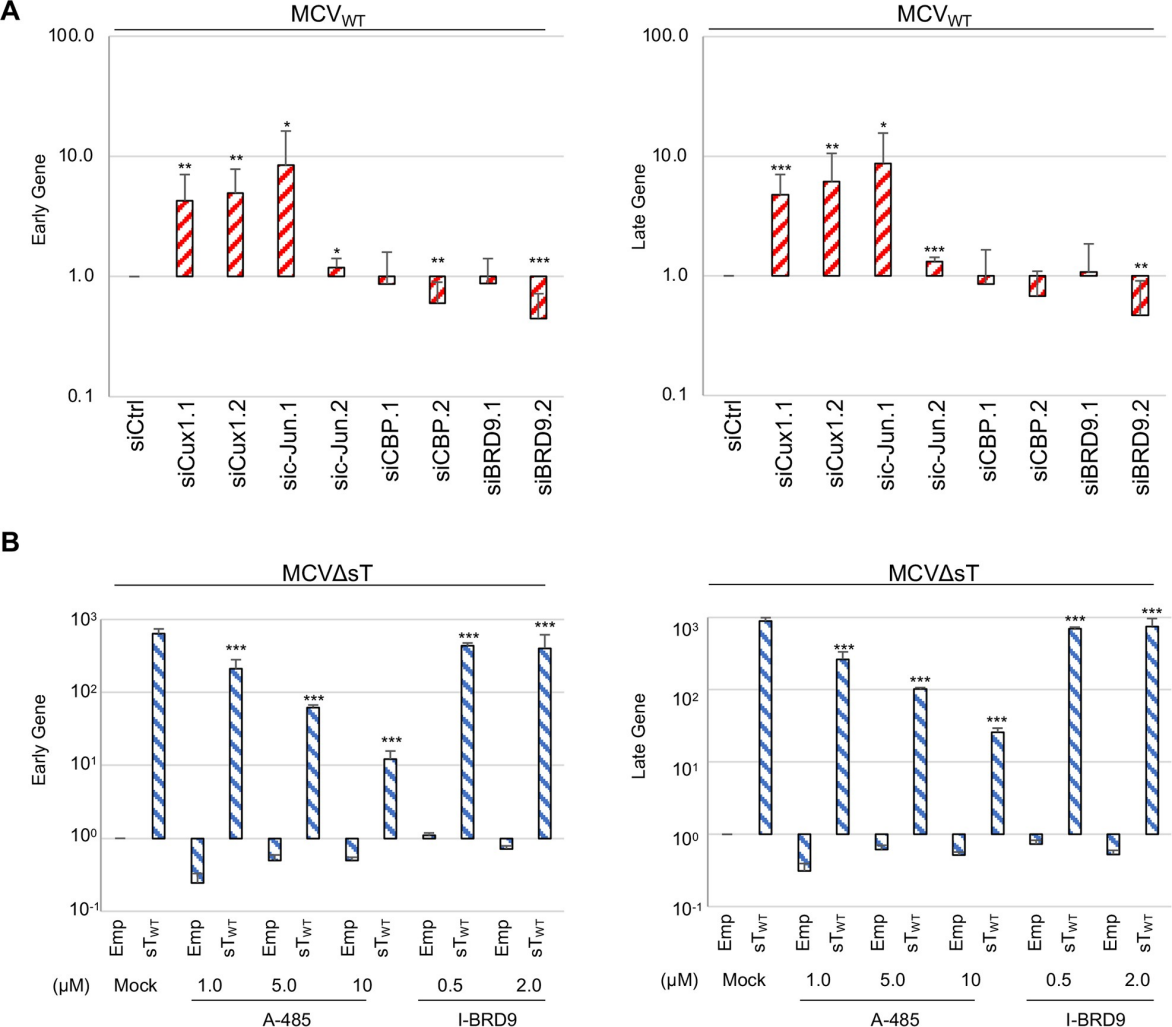
Role of sT-interacting proteins in MCV transcription and viral replication. **(A)** Effect o**f** siRNA knockdown of sT interactors on viral gene expression in MCV_WT_. siRNA treatment of MCV_WT_ samples. In cells treated with siCux1.1, siCux1.2, sic-Jun.1, and sic-Jun.2, early and late gene expression increases relative to the siCtrl. In cells treated with siCBP.2 and siBRD9.2, early and late gene expression decreases. Total RNA was subjected to qRT-PCR analysis by the 2^-ΔΔCt^ method using VP2 (set 1) and PanT primer pairs with 18S ribosomal RNA for normalization. Error bars indicate SD. Unpaired T-test was used for statistical analysis to determine the significance relative to siCtrl. *, P<0.05; **, P<0.01; ***, P<0.001. **(B)** A-485 and I-BRD9 treatment of MCVΔsT transfected with empty or sT. A-485 treatment significantly decreases early and late gene expression at 1.0, 5.0, and 10.0 μM in MCVΔsT transfected with sT. I-BRD9 treatment at 0.5 and 2.0 μM also significantly decreases early and late gene expression. Total extracted RNA was subjected to qRT-PCR analysis as described in Fig 8A. Relative mRNA expression to the sT_WT_-transfected sample is shown. Error bars indicate SD. Unpaired T-test was used for statistical analysis. *, P<0.05; **, P<0.01; ***, P<0.001.

To study the importance of the identified sT interactors in MCV transcription, we knocked down the four aforementioned transcriptional regulators and examined their effect on viral early and late gene expression. MCV_WT_ was co-transfected with two siRNAs, each with different targeting sequences, that knock down either Cux1, c-Jun, CBP, or BRD9 in 293 cells. Early and late gene expression was evaluated at day 4 p.t.. We achieved over 80% knockdown efficiency as quantitated by LI-COR immunoblot except for siCux1.1 (77%), sic-Jun.2 (63%), and siCBP.1(53%), which partially inhibited target protein expression (**S6A Fig**). Knockdown with siCux1.1, siCux1.2, and sic-Jun.1 increased early (4.3-, 4.9-, 8.4-fold, respectively) and late gene (4.8-, 6.1-, 8.7-fold, respectively) expression, suggesting that these transcriptional regulators may be negative regulators of viral transcription **(Fig 8A)**. sic-Jun.2 showed a moderate increase in early (1.2-fold) and late gene (1.3-fold) expression, consistent with lower knockdown efficiency. On the other hand, knockdown with siCBP.2 and siBRD9.2 decreased early (0.6- and 0.4-fold) and late (0.7- and 0.5-fold) gene expression. We did not observe significant effects in siCBP.1 due to low knockdown efficiency. These results suggest that CBP1 and BRD9 may act as transcriptional activators (**Fig 8A**).

Since siRNA knockdown was only partial and knockdown phenotype varied between the two siRNAs used for CBP and BRD9, we next tested if small molecule inhibitors that target CBP and BRD9 would affect sT-induced MCV transcription. For CBP inhibition, we used A-485, a potent and specific CBP/P300 histone acetyltransferase inhibitor shown to inhibit H3K18 acetylation [29,30]. To examine the effect of the inhibitor on sT-induced viral transcription, 293 cells co-transfected with MCVΔsT and sT_WT_ or an empty vector were treated with A-485 for 4 days p.t.. A-485 treatment significantly reduced sT-induced viral gene expression in a dose-dependent fashion while, in the absence of sT, no dose-dependent effect was seen despite less reduction (**Fig 8B**). At the highest dosage, 10 μM, early and late gene activation induced by sT (633.5-fold and 885-fold, respectively) from the MCVΔsT genome was attenuated by >97% (12.2-fold and 25.9-fold). For BRD9 inhibition, we used I-BRD9, a structure-based inhibitor that specifically binds BRD9. At concentrations of 0.5 and 2.0 μM, I-BRD9 led to decreased panT and VP2 expression by approximately 25% in MCVΔsT cells transfected with sT_WT_, yet the magnitude of reduction was not as large as that seen with A-485 treatment **(Fig 8B).** We also tested the effects of A-485 and I-BRD9 on MCV DNA replication by MCV origin replicon assays **(S6B and S6C Fig)**. sT-mediated replication activation by LT stabilization was not inhibited by either A-485 or I-BRD9, indicating that these inhibitors do not affect LT stabilization by sT and DNA replication from the viral origin. I-BRD9 demonstrated a dose-dependent increase in sT-induced replication activity up to 2.7-fold at 2.0 μM. Therefore, the significant reduction in early and late gene expression following inhibition of CBP and the modest reduction following inhibition of BRD9 strongly suggest that both CBP and BRD9 interact with sT to regulate MCV gene expression, but not DNA replication.

**Fig 9:**
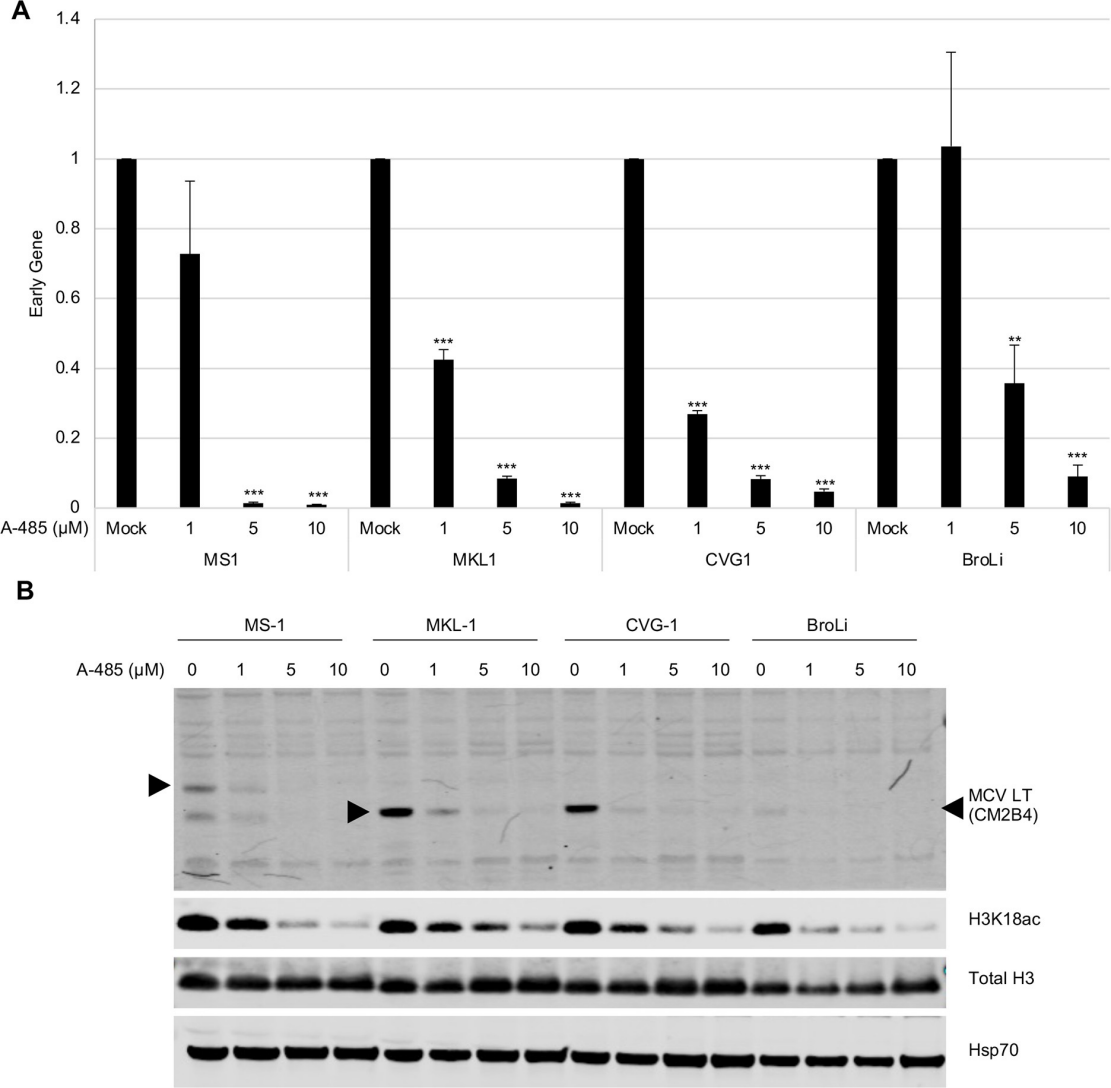
CBP regulates T antigen expression in MCV-positive MCC. **(A)** CBP/p300 inhibitor, A-485, significantly decreases early gene expression. MCV-positive CVG-1, MKL-1, MS-1, and BroLi cell lines were treated with various concentrations of A-485 and harvested at day 4. Total extracted RNA was subjected to qRT-PCR analysis by the 2^-ΔΔCt^ method using the PanT primer with 18S ribosomal RNA for normalization. Relative mRNA expression to the mock-treated sample is shown for each respective cell line. Error bars indicate SD. Unpaired T-test was used for statistical analysis. *, P<0.05; **, P<0.01; ***, P<0.001. **(B)** Corresponding immunoblots for qRT-PCR shown in Fig 9A. MCV LT was detected by CM2B4. H3K18ac and total H3 were detected to confirm A-485 activity. HSP70 was used as a loading control.

Since CBP inhibition substantially decreased MCV early and late gene expression, we next tested the effect of inhibiting CBP on early gene expression in four MCV-positive MCC cell lines **(Fig 9A)**. After treatment with 1.0, 5.0, and 10.0 μM of A-485, panT expression significantly decreased in MS-1, MKL-1, and CVG-1 cell lines relative to their respective mock-treated samples. At 10 μM, panT expression decreased by approximately 98% in MS-1, 98% in MKL-1, and 95% in CVG-1. In the BroLi cell line, a significant decrease in early gene expression occurred beginning at 5 μM, as compared to 1 μM in the other three cell lines, suggesting that BroLi may be more resistant to A-485 treatment. Corresponding immunoblots further confirmed decreased LT protein expression with increasing A-485 drug concentration in all four cell lines, further suggesting dose-dependency **(Fig 9B)**. Total H3 and H3k18ac were also measured as a control for A-485-mediated CBP inhibition since H3K18 is one of the major CBP/P300 acetylation sites [31]. H3K18ac protein expression decreased with increasing A-485 concentration, while total H3 expression remained constant, which confirms dose-dependent inhibition of CBP/P300.

EP400/NuA4/Tip60 histone acetyltransferase complex, a known interactor of sT, is another epigenetic transcriptional regulator that may contribute to viral transcription and replication. To test this possibility, we performed an MCV origin replication assay with EP400 and three LTSD mutants to test their MCV origin replication activities **(S7A Fig)**. The sT_90-95A_ mutant that was originally identified as an EP400-binding mutant did not activate MCV origin replication, similar to sT_LTSD_ and sT_90-94A_. However, two other EP400 mutants, 83-88A and 4M, stabilized LT and activated MCV origin replication. An MCVΔsT replication rescue assay with co-expression of the EP400 mutants demonstrated that, like sT_LTSD_, the sT_90-95A_ mutant failed to restore MCVΔsT replication, consistent with continued LTSD control of MCV replication. On the other hand, the sT_83-88A_ and sT_4M_ mutants rescued replication (**S7B Fig**). As demonstrated by Southern blot (**S7C Fig**), when we transfected the MCV genome with EP400 binding mutations, the MCV_sT90-95A_ mutant showed reduced replication like that seen in MCVΔsT. Replication of MCV_sT4M_ was also moderately decreased. We also examined the importance of EP400/Tip60 in MCV mRNA expression by knocking down EP400 in MCV_WT_ transfected cells. Contrary to our prediction, shRNA knockdown of EP400 did not inhibit early and late gene expression, but rather increased expression (**S6D and S6E Fig**). These results are consistent with the fact that EP400/Tip60 targeting of sT inhibits MCV replication and gene expression despite its important role in sT’s transformation activity [20].

## Discussion

The role of sT in the polyomavirus lifecycle is underrepresented in previous SV40 studies, likely because (1) LT is sufficient to initiate viral DNA replication and sT is dispensable *in vitro* [32], and (2) deletion of sT in SV40 does not ablate viral DNA replication and only attenuates replication [8]. While sT is not essential for viral DNA replication, the replication stimulatory function of sT is also seen in murine polyomavirus (PyV), as demonstrated by a report showing that reduced replication in PyV lacking both sT and middle T can be partially rescued by sT expression [33]. Due to the absence of sT’s stimulatory effects in *in vitro* replication assays, sT is suspected to affect viral replication indirectly by creating an optimal cellular environment for viral replication. A study with SV40 sT mutants demonstrated that sT-PP2A interaction is critical for rescuing the reduced replication of sT-deleted SV40 [9], suggesting that PP2A inhibition is an essential element of sT’s replication stimulatory function in SV40. The importance of sT-PP2A interaction in viral DNA replication has also been shown in JCV [10].

The importance of sT in MCV replication has been demonstrated by MCV replicon assays in which plasmid containing the viral replication origin was co-transfected with sT and LT expression vectors, replicating the artificial plasmids. Activation of the viral origin replication is dependent upon the stabilization of LT, which is mediated by sT’s inhibitory effect on host E3 ligases that degrade LT [11]. Furthermore, the domain responsible for large T stabilization, the LTSD, is important for both cell transformation and viral replication. However, another similar assay suggested that sT-induced LT stabilization is cell line-dependent, and that sT can activate origin replication without stabilizing LT [34]. Although sT activates MCV origin replication in an LT-dependent mechanism, sT may also exploit other mechanisms to activate viral origin replication.

**Fig 10:**
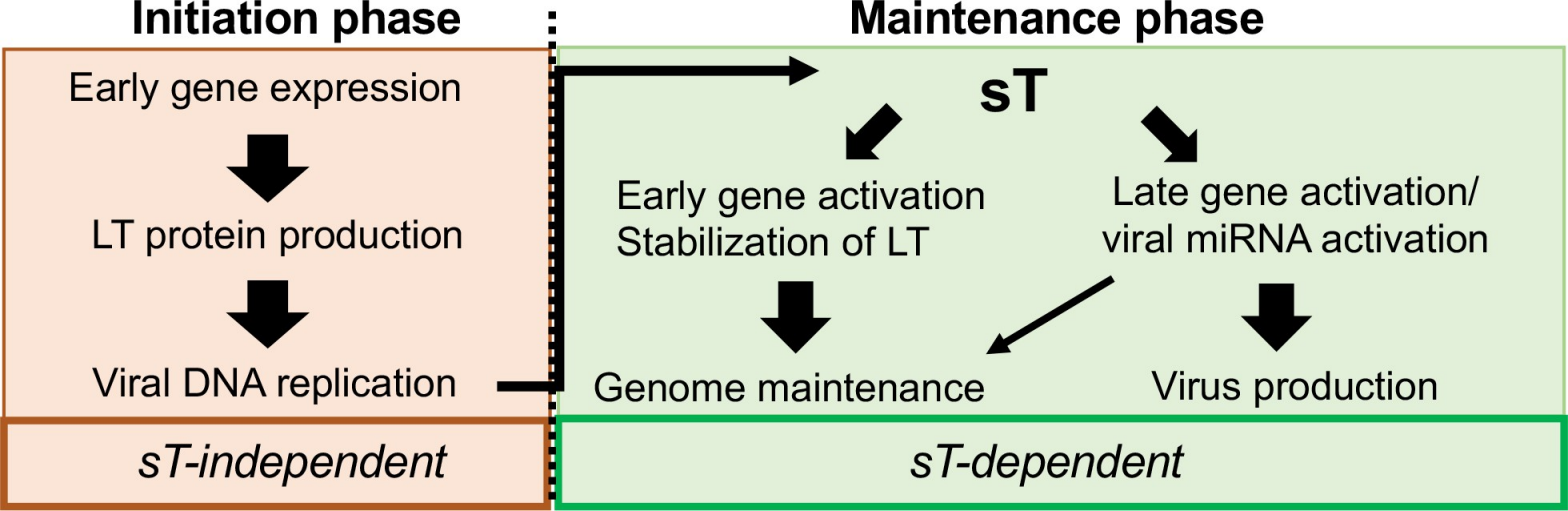
Importance of two MCV replication phases for sT function. Replication initiation phase, including early gene expression, LT production, and initial viral origin replication, does not require sT function, but activates sT. In the maintenance phase of replication, sT regulates viral transcription targeting factors such as Cux1, c-Jun, and CBP as well as DNA replication by stabilizing LT [11]. Maintenance of LT stability and early gene expression may control genome maintenance. Late gene induction that results in viral capsid assembly progresses viral life cycle and may also control genome maintenance via viral miRNA expression [21].

While the importance of sT in origin replication was apparent, the role of sT expressed from the native MCV genome remained unclear. In this study, our results with MCVΔsT, a mutant lacking the entire sT gene suggest that while sT is dispensable for initiation of viral DNA replication, it is critical for activating viral DNA replication and viral gene expression after initial replication to maintain the viral genome and promote viral life cycle progression. Furthermore, our observation that sT could not activate viral gene expression in the presence of DNA replication inhibitors indicates that prior viral DNA replication is a prerequisite for MCV gene activation by sT. Based on these findings, we propose two MCV replication phases upon entry of the viral genome into the nucleus: (1) an initiation phase requiring only LT expression and (2) a maintenance phase depending on both LT and sT, in which sT controls early and late gene expression to advance the viral lifecycle and maintain LT protein levels (**Fig 10**). We also found that the functions of sT in MCV replication are exerted through the LTSD, a previously defined domain that is critical for LT stabilization, activation of viral origin replication, and malignant cell transformation [11]. Taken together with previous findings, our results suggest that, throughout MCV infection and MCC carcinogenesis, sT remains functionally active to maintain viral persistence and carcinogenesis.

Interestingly, despite its importance in SV40 and JCV, sT-PP2A interaction was dispensable for MCV sT’s replication and transcription stimulatory functions. Thus, while the replication stimulatory effect of sT is seen across polyomaviruses, MCV sT exploits a different mechanism to promote viral replication and transformation.

Although sT is an important protein in the MCV lifecycle and transformation, sT protein levels were found to be low in MCV. Thus, we exploited codon-optimized sT protein for functional studies to achieve protein expression detectable by antibody staining in the majority of the experiments. The sT protein is localized in both the cytoplasm and the nucleus, whereas the full-length LT protein predominantly localizes in the nucleus. It is likely that sT interacts with various cytoplasmic and nuclear proteins to regulate viral replication and cell transformation. Our immunofluorescence results demonstrate that mutations in the LTSD reduced nuclear expression of sT in transfected 293 cells. This result is consistent with the importance of nuclear sT protein in viral genome maintenance and suggests a possibility that the LTSD alters nuclear sT protein stability or sT localization. It should be noted that LTSD mutation is less likely to affect global protein structure because both wild type sT and LTSD mutant bind to the PP2Ac protein, and profiles of proximal host proteins to wild type and LTSD were similar, as shown by Bio-ID.

Our Bio-ID assay with sT_WT_ and sT_LTSD_ as bait proteins, successfully catalogued cellular proteins physically localized near sT as the “proxisome”. Although the protein-protein interactions identified by proximity-based assays do not warrant physical protein-protein interactions, our IP experiments confirmed the interaction of sT with multiple transcriptional regulators identified by Bio-ID. Aside from the transcriptional regulators extensively analyzed in this study, we identified larger functional groups of proteins as MCV sT’s proxisome. These include nucleic acid metabolism proteins, metabolite interconversion enzyme, cytoskeletal proteins, protein modification enzymes, and translational proteins.

We further studied specific subclasses of the four largest groups identified by PANTHER analysis of the Bio-ID proteins **(**S4B **Fig).** While direct protein interaction between these protein groups and sT is not confirmed, these proteins are closely associated with known sT phenotypes including increased mitogenesis and destabilization of microtubules [35,36]. Interestingly, 16% of proteins identified in the cytoskeletal group belonged to the microtubule subclass, consistent with sT’s potential interaction with microtubules to regulate mitogenesis. The potential interaction between sT and RNA splicing factors, RNA metabolism factors, general transcription factors, oxidoreductases, and microtubules may be important to the function of sT. Polyomavirus late mRNA expression is regulated at the level of RNA splicing and RNA stability by a unique splicing called leader-to-leader splicing that occurs from polyA-read through late transcripts [21–23,37,38]. Thus, splicing factors and metabolism factors for RNA, which comprise 28% and 18% of nucleic acid metabolism proteins, respectively, may be important targets of sT for MCV mRNA expression. General transcription factors, which comprise 12% of nucleic acid metabolism proteins, may interact with sT to regulate early and late gene expression. Oxidoreductases, which comprise 44% of metabolite interconversion enzymes and are involved in the mitochondrial electron transport chain, may also contribute to sT function as sT has previously been shown to activate anaerobic glycolysis [39] although a direct effect on oxidative phosphorylation was not observed. Additional studies may confirm the involvement of these protein groups in sT’s MCV replication activity.

Immunoprecipitation identified four transcriptional regulators that partially bind to sT in the nucleus in 293 cells: p200-Cux1, c-Jun, CBP, and BRD9. Interestingly, Cux1 and c-Jun not only co-localized with sT, but also with LT foci in the nucleus, the site of MCV replication. This suggests that these transcription factors may directly control viral gene expression. Furthermore, loss-of-function studies with siRNA suggest that Cux1 and c-Jun are negative regulators of MCV early and late gene expression. *Cux1* produces multiple isoforms including three nuclear Cux1 proteins with a DNA binding domain, p200-Cux1, p110-Cux1, and p75-Cux, and a cytoplasmic Cux1 protein called CASP, localized in the Golgi apparatus [40]. Our antibody detected only p200-Cux1 and CASP proteins. While the cytosolic function of CASP remains unknown, nuclear full-length p200-Cux1 is a transcription factor that negatively regulates gene expression in various viruses including HPV type 6 [41], HPV type 16 [42], retrovirus, and cytomegalovirus [43,44]. These reports indeed support a negative regulatory function of p200-Cux1 in MCV replication. The transcription factor c-Jun is part of the AP-1 family of transcription factors, which control various cellular and viral promoters and regulate cellular and viral processes such as cell death, cell proliferation, and differentiation [45]. In fact, AP-1 was first discovered as a TPA-activated transcription factor that binds specific sequences in the 72 bp repeat of the SV40 enhancer region [46]. The AP-1 consensus sequence can also be found in the JCV promoter [47], which activates early and late gene transcription. We confirmed the presence of the AP-1 consensus sequence in the MCV NCCR. Cux1 and c-Jun are promising candidates that could regulate MCV transcription. Further biochemical and functional analyses are required to understand the role of sT interaction with Cux1 and c-Jun in MCV transcription.

We also identified CBP, a histone acetyltransferase that regulates transcription, as a positive regulator of MCV gene expression. Interestingly, MCV sT binds to the EP400-Tip60 HAT complex and MycL to promote MCC carcinogenesis [20]. Tumor virus oncoproteins, such as SV40 LT and adenovirus E1A, also bind to both CBP/EP300 and EP400, contributing to viral replication and cell transformation [48–50]. Our results suggest that CBP/EP300 positively regulates viral transcription, whereas EP400 negatively controls viral transcription. Similar results have been shown in the HPV18 virus [51,52]. Since sT recruits the EP400/Tip60 HAT complex for Myc-L/Max transcription on the host chromatin in MCC [20], sT may recruit CBP to activate other transcription factors on viral chromatin. However, whether or not sT and these transcriptional regulators bind to the chromatinized viral NCCR and directly control viral gene expression is uncertain. Further studies are necessary to elucidate the functional consequence of sT interaction with these transcription factors in viral gene expression and genome maintenance.

Since MCV infection causes MCC through persistent infection, understanding the molecular mechanism of MCV persistence may provide important clues towards the prevention of MCC. Together with previous findings on SV40 sT in mesothelial cells [9], our study suggests that sT plays a role in viral genome maintenance by promoting viral DNA replication and gene transcription. As previously reported, sT may regulate viral persistence by fine-tuning LT replicase protein levels [12]. In addition, viral miRNA MCV-miR-M1 has been shown to regulate viral persistence [21]. MCV-miR-M1 is encoded by the negative strand of the T antigen gene and produced from read-through late gene transcripts or transcripts driven by the non-canonical promoter within the T antigen gene [21,53]. Since sT is essential for viral late gene activation, it is likely that sT also upregulates MCV-miR-M1 to adjust viral early gene expression for long-term maintenance of the viral episome (Fig 10).

We found that the MCV sT oncoprotein, which has transformation activity *in vivo* and *in vitro*, controls viral persistence and viral T antigen expression in MCV-positive MCC tumors by targeting the shared transcriptional regulator CBP. Since MCC tumorigenesis is addicted to viral T antigen expression, further mechanistic analysis of viral transcription could lead to the development of novel chemotherapeutics to disrupt viral persistence and, ultimately, MCC carcinogenesis.

## Materials and methods

### Cell lines, cell culture, and transfections

Cell lines that were used include: HEK293, BJ.hTERT, MKL-1, MS-1, CVG-1, and BroLi. HEK293 and BJ.hTERT cells were cultured in Dulbecco’s modified Eagle’s Medium (DMEM) while MKL-1, MS-1, CVG-1, and BroLi cells were cultured in RPMI media. All media was supplemented with 10% Fetal Bovine Serum (FBS) and 5 mL of Non-Essential Amino Acids (NEAA). Cells were kept at 37°C and 5% CO_2_ in an incubator. Cells were tested for mycoplasma throughout the experiments to confirm negativity. For plasmid DNA transfections, 293 cells/293 TRE cell lines were transfected at 40–50% density using Lipofectamine 2000 (Invitrogen). To screen active siRNAs, Lipofectamine RNAiMAX transfection (Invitrogen) was used. All transfection reagents were used according to manufacturer’s instructions.

### Plasmids

A full-length wild type MCV-HF genome (GeneBank accession no. JF813003) and MCV-HF with a rep- mutation (cytidine 44 to adenine in JF813003) were cloned into pSMART-LC-Amp cloning vector (Lucigen) to generate pSMART MCV-HF and pSMART MCV-HF_rep-_. This was done using an EcoRI site that digests once in the MCV genome as described previously [16,18]. To construct sT mutants on pSMART MCV-HF (ΔsT, MCVsT_83-88A_, MCVsT_90-95A_, MCVsT_4M_, MCVsT_LTSD_, MCVsT_90-94A_), we used overlapping PCR. First, PCR was performed using AvrII.MCV.NCR.F with a mutagenic reverse primer, and a corresponding mutagenic forward primer with BamHI.R **[S2 Table]**. The two PCR products containing overlapping sequences were purified by PCR/Gel purification columns (Thomas Scientific) and overlapping PCR was performed with AvrII.MCV.NCR.F and BamHI.R primers containing AvrII and BamHI restriction sites **[S2 Table]**. The PCR-ligated product was then cloned into pSMART MCV-HF using the unique AvrII and BamHI sites.

Using a similar strategy, MCV encoding ZsGreen (ZsG) under the late promoter was generated by inserting a ZsG-FMDV 2A sequence before the VP2 initiation codon (S2A **Fig**). A ZsG-FMDV 2A fragment synthesized ZsG cDNA (IDT) using VP2.NCR.ZsGreen.F and 2A.VP2.R. Two MCV fragments were also amplified from pSMART MCV-HF using AvrII.MCV.NCR.R and VP2.NCR.R, or 2A.VP2.F and VP2.PacI.R **[S2 Table]**. Three overlapping PCR fragments mixed with equal molar ratios were fused by PCR using AvrII.MCV.NCR.R and VP2.PacI.R. The PCR-ligated fragment was then cloned into pSMART MCV-HF, pSMART MCV-HF_rep-_, pSMART MCVΔsT, and pSMART MCVΔsT_rep-_ with unique AvrII and PacI sites. pcDNA MCV LT, a codon-optimized MCV LT expression vector, and codon-optimized MCV sT expression vectors pcDNA6 MCV sT_WT_, pcDNA6 MCV sT_LTSD_, pcDNA6 MCV sT_D44N_, pcDNA6 MCV sT_R7A_, pcDNA6 MCV sT_L142A_, pcDNA6 MCV sT_D44N_, pcDNA6 SV40 sT_WT,_ and pcDNA6 SV40 sT_L133A_ were previously generated [5,6]. Additional mutants including pcDNA6 MCV sT_83-88A_, pcDNA6 MCV sT_4M_, pcDNA6 MCV sT_90-95A_, and pcDNA6 MCV sT_90-94A_ were generated by the QuickChange site-directed mutagenesis kit (Agilent) using primers in **S2 Table**.

Lentivirus constructs that inducibly express codon-optimized sT (pLenti TRE-MCV sT) were described previously [54]. To generate an inducible lentivirus vector expressing MCV LT, a codon-optimized LT fragment digested from pcDNA6 MCV LT with KpnI and XhoI was cloned into pENTR1A. The resulting pENTR1A MCV LT was then LR-recombined into pTRE.DEST EF.Puro-rTet [55] to generate pLenti TRE-MCV LT by LR clonase (Invitrogen).

For the proximity ligation assay, we constructed a parental lentivirus vector that inducibly expresses a C-terminally BirA-tagged fusion protein. BirA (R118G) cDNA was amplified by primers containing KpnI.linker.BirA.F and BamHI.BirA.R sites **[S2 Table]**, and the resulting fragment was cloned into the pLenti TRE-MCS EF.Puro-2A-rTet vector [54] with KpnI and BamHI restriction sites to generate pLenti TRE-MCS-BirA EF.Puro (Emp-BirA). Codon-optimized sT_WT_ and the sT_LTSD_ mutant (91-95A) fragments were amplified from pcDNA6 MCV sT_WT_ and pcDNA6 MCV sT_LTSD_ using AfeI.sTco.F and KpnI.sTco.R **[S2 Table]**, and then cloned into pLenti TRE-MCS-BirA EF.Puro using AfeI and KpnI sites to produce pLenti TRE-sT_WT_-BirA EF.Puro (sT_WT_-BirA) and pLenti TRE-sT_LTSD_-BirA EF.Puro (sT_LTSD_-BirA), respectively.

To construct pcDNA3 vectors that express codon-optimized sT fused to the C- and N-terminus Strep-II-FLAG epitope (SF) tag, pcDNA3 CSF-empty and pcDNA3 NSF-empty vectors were used [56]. Codon-optimized sT cDNA fragments were amplified by two primer sets containing NheI and XhoI restriction sites for the NSF tag, and KpnI and NotI restriction sites for the CSF tag **[S2 Table]** from pcDNA6 MCV sT_WT_ and pcDNA6 MCV sT_LTSD_. Next, the wild type and LTSD mutant sT cDNA were cloned into pcDNA3 CSF-empty and pcDNA3 NSF-empty vectors using corresponding unique sites (NheI and XhoI or KpnI and NotI). CSF-empty, CSF-sT and NSF-sT fragments were then transferred to the pENTR 1A vector using unique KpnI and XhoI sites. The resulting pENTR 1A vectors encoding CSF-empty, CSF-sT_WT_, CSF-sT_LTSD_, NSF-sT_WT_, NSF-sT_LTSD_ were LR-recombined into pTRE.DEST EF.Puro-rTet to generate pLenti TRE-CSF-empty, pLenti TRE-CSF-sT_WT_, pLenti TRE-CSF-sT_LTSD_, pLenti TRE-NSF-sT_WT_, pLenti TRE-NSF-sT_LTSD_ by LR cloning (Invitrogen). All sequences were confirmed by Sanger sequencing (MCLAB).

### Antibodies

Primary antibodies include: Cux1 (11733-1-AP, Proteintech), BRD9 (24785-1-AP, Proteintech), c-Jun (24909-1-AP, Proteintech), CBP (7389S, Cell Signaling), DYKDDDDK Tag (FLAG) (A00187S, Genscript), Total H3 (14269, Cell Signaling), H3K18Ac (13998, Cell Signaling), Anti-SV40 T antigen PAb419 (sc-58665, Santa Cruz), and Hsp70 (sc-7298, Santa Cruz). Primary antibodies against MCV LT (CM2B4, VP1, CM9B2) were gifted from Dr. Patrick Moore and Dr. Yuan Chang’s laboratory. 2T2 was gifted from Dr. Christopher Buck’s laboratory. Secondary antibodies for immunoblots include: goat anti-rabbit IRDye 800CW (926–32211, LI-COR), goat anti-mouse IRDye 800CW (926–32210, LI-COR), IRDye 800CW Streptavidin (926–32230, LI-COR). Secondary antibodies for immunofluorescence include: Alexa488 anti-mouse (Invitrogen), Alexa568 anti-mouse (Invitrogen), Alexa488 anti-rabbit (A11034, Invitrogen), Alexa568 anti-rabbit (A11036, Life Technologies).

### Genomic and episomal DNA preparation

Cell pellets were suspended overnight in lysis buffer (10 mM Tris, 100 mM NaCl, 0.5% SDS, 25 mM EDTA, pH 8.0) with 100 μg/mL of proteinase K at 37°C. 100 μg/mL RNase was then added for 1 h at 37°C. After lysis, genomic DNA was acquired by phenol chloroform and ethanol precipitation. To extract episomally replicating DNA, transfected cells were harvested and lysed in 600 μL of 0.6% SDS, 10 mM Tris, 1 mM EDTA, pH 8.0. 100 μL of 5M NaCl was added to 400 μL of the lysate and incubated overnight at 4°C. Samples were then centrifuged at 13,000 rpm for 30 min at 4°C to separate the supernatant from the insoluble fraction. The supernatant was subsequently removed and subjected to phenol chloroform and ethanol precipitation to acquire episomal DNA.

### Re-circularization of MCV-HF clone

Wild type and mutant pSMART MCV-HF plasmids were digested with EcoRI overnight at 37°C followed by EcoRV for 5 h at 37°C. This reaction produces a full-length linear MCV-HF genome with self-ligatable EcoRI DNA ends and a pSMART-LC-Amp vector fragment with EcoRV ends. The digested sample was purified using plasmid purification columns (Thomas Scientific) to acquire linearized MCV DNA. Re-circularization of the MCV-HF genome was performed by the T4 DNA ligase system (NEB) overnight at 16°C. To prevent concatenation of the MCV genome, the ligation reaction was performed at a lower DNA concentration (2.5 ng/μl) overnight. The re-circularized MCV genome was purified using the same plasmid purification columns. DNA eluted with 10 mM Tris pH 8.0 was used directly for transfection experiments without removing the pSMART vector fragment.

### Total RNA extraction, DNase treatment, and cDNA synthesis

Total RNA was extracted by the JET RNA Purification Kit (Thermo Scientific). Acquired RNA was then treated with the Invitrogen TURBO DNA-free Kit (Invitrogen). Following DNase treatment, the RNA was converted to cDNA by random hexamers using the iScript cDNA synthesis Kit (Bio-Rad). To detect the positive strand of the MCV early gene, cDNA was synthesized by a T antigen specific primer (Pan T2) using the Super Script IV cDNA synthesis kit (Invitrogen). To confirm the amount of input RNA, 18S rRNA expression was detected from the cDNA synthesized by random hexamers.

### Quantitative PCR

Genomic DNA was digested with DpnI at 37°C overnight and purified using plasmid purification columns to remove non-replicating DNA (Thomas Scientific). 20 ng of DNA per sample was used for qPCR using the PowerUp SYBR Green Master Mix Kit (Applied Biosystems). For qRT-PCR, cDNA reverse transcribed from 25 ng equivalent of total RNA was used for each sample and cDNA reverse transcribed from 10 ng equivalent of total RNA was used for the 18S control. qPCR was performed using QuantStudio 3 (Applied Biosystems). Data was obtained using the ΔΔCT method. Primers used for qPCR and qRT-PCR are found in **S1 Table**.

### Southern blot hybridization

gDNA was digested with EcoRI and DpnI overnight. 5–8 μg of DNA was loaded in a 0.8% agarose gel. The DNA ladder used was a 1kb Plus DNA ladder (NEB, N3200S). Equal loading and full digestion of DNA was checked with ethidium bromide staining. DNA was then transferred with 10X SCC onto a nitrocellulose membrane overnight and DNA was then cross-linked onto the membrane. To generate an MCV probe, MCV-HF genome that was excised from pSMART MCV-HF with BsrFI was biotinylated by using the BioPrime Array CGH kit (Invitrogen). The membrane was hybridized with the MCV probe at 65°C overnight in a hybridization buffer (5X SSPE, 2% SDS, 10% dextran sulfate, 1X Denhardt’s Solution, 10 μg/mL sonicated salmon sperm DNA). Hybridization signal was detected by Streptavidin-IRDye 800CW (LI-COR) and replication was detected by LI-COR imager.

### Immunoblotting

Harvested cells from each experiment were suspended in RIPA buffer with a proteinase inhibitor cocktail (Roche Complete Tablet) and sonicated until cell membranes were fully disrupted to release protein. The protein was then quantified using BSA (bovine serum albumin) standards on a Biotek Synergy 2 machine. After quantification, samples were mixed with NuPAGE LDS Sample Buffer 4X (Invitrogen) to make a 1X concentration with 5% beta-mercaptoethanol. Protein was loaded in an SDS-PAGE gel with percentages adjusted to detect the target protein of interest. Precision Plus Protein Kaleidoscope (Bio-Rad) was used as a ladder for all immunoblots. After running the gel, protein was transferred onto a nitrocellulose membrane overnight using a wet transfer buffer at 30 V. The membrane was blocked for 30 min in 5% skim milk in TBS at room temperature. Primary antibodies were diluted either in 5% BSA or 5% skim milk in TBS and incubated overnight at 4°C, or 5 h at room temperature. Membranes were washed 3 times in TBS-T. Secondary antibodies (anti-mouse or anti-rabbit) were diluted in 5% skim milk in TBS and membranes were incubated for 1 h at room temperature in the absence of light. Membranes were washed again in TBS-T, and lastly imaged on a LI-COR imager for analysis.

### Proximity-based biotin labelling and purification of biotinylated proteins

293 TRE-Emp-BirA, sT_WT_-BirA, and sT_LTSD_-BirA cells were seeded in five 14.5 cm dishes at 5x10^6^ cells per dish. Five μg of MCVΔsT re-circularized DNA was transfected per dish 24 h after seeding by Lipofectamine 2000. Cells were supplemented with doxycycline 24 h p.t. at 0.5 μg/mL. At 72 h p.t. doxycycline was refreshed, and biotin was added at 50 μM. 24 h following biotin addition, cells were washed in PBS, resuspended in SDS lysis buffer (1% SDS, 10 mM EDTA, 50 mM Tris HCl, 1 mM PMSF, pH 8.0) with protease inhibitor cocktail (Roche), and sonicated to acquire the soluble protein fraction. Total biotinylated proteins from sT_WT_-BirA, sT_LTSD_-BirA, and Empty-BirA were purified by Dynabeads MyOne Streptavidin C1 (Invitrogen) for mass spectrometry.

### Mass spectrometry

Mass spectrometry protocol for Bio-ID experiments was previously described [57]. Briefly, samples were heated at 85°C for 5 min, separated ~1.5 cm on a 10% Bis-Tris Novex mini-gel (Invitrogen) with the MES buffer system, and stained with coomassie. This process separated lanes into 10 fragments of the same size, which were then reduced using dithiothreitol, alkylated with iodoacetamide, and digested with trypsin (Promega, Wisconsin). The digested samples were then subjected to Nano LC-MS/MS with a NanoAcquity HPLC system (Waters, Massachusetts) interfaced to a Fusion Lumos tandem mass spectrometer (ThermoFisher, California) using a 3 s cycle. Using a trapping column with Luna C18 resin (Phenomenex, California), peptides were eluted at a rate of 350 nL/min. A 30 min gradient was employed for each segment. Product ion data were searched against the combined forward and reverse Swissprot *H*. *sapiens* protein database using a locally stored copy of the Mascot search engine v2.6 (Matrix Science, London, U.K.) via Mascot Daemon v2.6. Proteome Discoverer v2.1 (ThermoFisher) was used to make peak lists. Search parameters were precursor mass tolerance 10 ppm, product ion mass tolerance 0.02 Da, 2 missed cleavages allowed, fully tryptic peptides only, fixed modification of carbamidomethyl cysteine, variable modifications of oxidized methionine, protein N-terminal acetylation and pyro-glutamic acid on N-terminal glutamine. Scaffold software v4.8 (Proteome Software) was used to read flat files (DAT) that were generated by Mascot search with a filter for 1% protein and peptide level false discovery rate and for detection of at least two unique peptides for each protein. Proteins with at least a 5-fold difference in spectral counts between the control Emp-BirA sample and either sT_WT_-BirA or sT_LTSD_-BirA were selected as proteins proximity biotinylated by either sT_WT_ or sT_LTSD,_ respectively. Proteins were classified as common to both sT_WT_ and sT_LTSD_ if both had a greater than 5-fold difference in spectral counts compared to Emp-BirA, but spectral counts between sT_WT_ and sT_LTSD_ were less than 5-fold different [58].

### Immunoprecipitation

293 TRE-SF-Emp, CSF- and NSF-sT_WT_, and NSF-sT_LTSD_ cells were seeded in 14.5 cm dishes and left to adhere for 24 h prior to doxycycline treatment to induce sT expression. After 72 h, cells were harvested from each plate and split into two pellets per dish. Protein was extracted using IP lysis buffer (25 mM Tris, 137 mM NaCl, 2 mM EDTA, 5% glycerol, 1% Triton X-100, pH 7.5) containing 0.5X protease inhibitor cocktail (Roche), 10mM NaF, and 10mM NaVO_3_. The pellet was rotated in 1 mL of IP lysis buffer for 10 min at 4°C before being re-pelleted, and the supernatant was taken for immunoprecipitation. 800 μL of the supernatant was used for IP while the remaining 200 μL was taken for input. Anti-FLAG antibody was added at a 2 μg/mL concentration rotating overnight at 4°C. 24 h following antibody addition, Dynabeads G (Invitrogen) were added, and samples were again rotated at 4°C for 90 min. Following the antibody reaction, beads were washed 4 times with IP lysis buffer containing 0.5 mM PMSF. The third wash contained 150 mM NaCl, in place of 137 mM, to decrease the background of immunoblots. After washing, 20 μL of 2X LDS sample buffer containing 10% 2-ME was added to each sample and denatured at 70°C for 10 min prior to loading. We used 2.5% of the total lysate for input. Samples were subjected to immunoblots.

### Immunofluorescence

BJ.hTERT TRE-Emp and TRE-sT cells infected with MCV_WT_ and MCV ΔsT were fixed with 10% buffered formalin for 8 min, permeabilized with 1X PBS containing 0.1% Triton X-100, blocked with 5% normal goat serum (NGS), and stained with anti-VP1 antibody (CM9B2, 1:2 dilution) for 3 h at room temperature. After washing with PBS, cells were treated with Fluor 488-conjugated anti-mouse IgG for 1 h (1:1000, Invitrogen). Antibodies were diluted in 5% NGS. For co-immunofluorescence staining in Figs 3B and S5D, 293 TRE-SF-Emp, NSF-sT_WT_, and NSF-sT_LTSD_ cells transfected with MCVΔsT were treated with Dox day 1 p.t., seeded on 12 mm coverslips day 3 p.t. and harvested day 4 p.t.. Cells on coverslips were fixed, permeabilized, and blocked as described above. Co-immunostaining was performed sequentially by probing one antigen after another by using mouse (FLAG at 1:500 dilution and CM2B4 at 1:100 dilution) and rabbit (Cux1, c-Jun, and CBP at 1:50 dilution) primary antibodies, which were detected with anti-mouse and anti-rabbit IgG conjugated with either Alexa Fluor 488 or Alexa Fluor 568 (1:1000 dilution, Invitrogen). Samples were reacted with primary and secondary antibodies for 1 h at 37°C and washed with PBS for 15 min before and after secondary antibody reaction. All antibodies were diluted in 5% NGS in PBS. Slides were mounted onto coverslips using DAPI Fluoromount-G (SouthernBiotech, Cat #0100–20). All staining was performed on IX81-DSU Spinning Disk Confocal Microscope (Olympus). The slides were scanned in z-stack mode, using 9 levels with an interplane spacing of 0.22 μm. A stack that included the sectioned nucleus with highest sT expression was subjected to pixel matching colonization analyses to determine Pearson’s correlation efficient by CellSens dimension software (Olympus). For FLAG sT, we used 293 TRE-SF Empty cells as a negative control, and for LT we used mock transfected cells as a negative control (S5A).

### Flow cytometry

MCV_WT._L-ZsG and MCV.L-ZsG_rep-_ as well as their corresponding sT deletion mutants (MCVΔsT.L-ZsG and MCVΔsT.L-ZsG_rep-_) were transfected into 293 cells and harvested 4 days p.t.. Cells were pelleted and fixed in 1.5 mL of 4% paraformaldehyde for 8 min. Cells were suspended in 1% FBS and analyzed using a Cytoflex LX (Beckman Coulter). To determine precise GFP positivity, autofluorescent cells were gated out by using FL1 and FL3 scatter plots by using the FCS 7.0 software (De Novo Software).

### siRNA and small molecule inhibitors

TriFECTa kits containing three Dicer-Substrate siRNAs for each target (Cux1, c-Jun, BRD9, and CBP) were purchased from IDT. Two of the best D-siRNAs were first screened by immunoblot. SignalSilence siRNA II targeting c-Jun was obtained from Cell Signaling. I-BRD9 and A-485 were purchased from Selleckchem Chemicals. DNA replication inhibitors aphidicolin and mimosine were purchased from Sigma, and used at 5 μM and 400 μM, respectively.

### Statistical analysis

Unpaired T-test was used for determining statistical significance for qRT-PCR analyses. Statistical significance was determined as follows: *, P<0.05; **, P<0.01; ***, P<0.001. qRT-PCR analyses were each repeated at least 3 times.

## Supporting information

S1 Fig LTSD of sT is critical for viral gene activation. **(A)** Stabilization of MCV LT by co-expression of sT_WT_ and various sT mutants. 293 cells co-transfected with LT and various MCV sT mutant expression vectors were harvested at 48 h p.t. and cell lysates were subjected to immunoblots with LT (CM2B4) and MCV sT (2T2) antibodies. Hsp70 was used to show equal protein loading. Three mutants, sT_LTSD_, sT_90-95A_, sT_90-94A_, that have mutations surrounding the LTSD (aa 90–95) failed to increase LT expression. While sT_90-95A_ is one of three EP400 binding mutants [20], two other mutants, sT_83-88A_ and sT_4M_, stabilized LT. MCV sT harboring mutations in DnaJ (D44N), PP2A (R7A and L142A), and both DnaJ and PP2A (D44N/L142A) increased LT to wild type levels. **(B)** 293 TRE-sT cells were co-transfected with MCVΔsT and pcDNA empty (Emp), and increasing concentrations of pcDNA sT_WT_ or LTSD mutants (sT_LTSD_, sT_90-95A_, and sT_90-94A_). Samples were harvested on day 4 p.t. RNA was extracted and converted to cDNA for qRT-PCR analysis to determine early and late gene expression as described in Fig 2B legend. Error bars represent SD. Regardless of concentration, LTSD mutants could not induce expression of the early or late gene as sT_WT_ does. **(C)** Replicated samples prepared as in S1B Fig were extracted for protein and used to determine sT, LT, and VP1 protein expression by immunoblot. Hsp70 was used as a loading control. Comparable protein expression is in red text. Even the smallest concentration of sT_WT_, 10 ng, can express LT and VP1, while the LTSD mutants cannot rescue LT or VP1, no matter their concentration.
(TIF)

S2 Fig Requirement of viral DNA replication for sT-mediated late gene reporter activation in MCV reporter viral genomes. **(A)** Generation of MCV late gene reporter viruses with or without sT deletion. MCV_WT_.L-ZsG and MCVΔsT.L-ZsG encoding the ZsGreen (ZsG)-FMDV-2A sequence before the VP2 coding sequence. 293 cells transfected with MCV_WT_.L-ZsG and MCVΔsT.L-ZsG with or without the rep- mutation were imaged under an inverted microscope at day 4 p.t. **(B)** Quantification of late gene-driven ZsG positive cells (top panel) and mean fluorescence intensity (bottom panel). Cells obtained from 6 independent transfections were analyzed by flow cytometry. sT deletion does not significantly decrease ZsG positive cells whereas introducing a mutation into the NCCR (MCVΔsT.L-ZsG_rep-_) decreased percent positivity by 50%. On the other hand, sT deletion reduced mean fluorescence intensity (MFI) by 43%. However, the rep- mutation cancelled the sT deletion effect and further attenuated MFI. Based on the decrease in ZsG positivity by the rep- mutation, ZsG positivity appears to be under the control of viral replication. In contrast, MFI may reflect late promoter activity since per-cell-intensity of ZsG was similarly lower in cells transfected with MCVΔsT.L-ZsG, MCV_WT_.L-ZsG_rep-_, and MCVΔsT.L-ZsG_rep-_ as in S2A Fig. **(C)** Day 4 p.t. samples treated with or without APC from Fig 4C and 4D were used to examine early gene mRNA expression. Total RNA was extracted and converted to cDNA using the Super Script IV and the PanT2 primer in S1 Table. PCR was performed using the same PanT primers as in Fig 4D. 18S ribosomal RNA was amplified from cDNA that was generated by iScript as a control.
(TIF)

S3 Fig MCV sT deletion attenuates viral gene expression in infected human fibroblast cells. **(A)** sT regulates VP1 expression in infected permissive cells. sT deletion in the MCV genome reduces early and late viral gene expression. BJ.hTERT cells infected with MCV_WT_ and MCVΔsT were harvested at various time points after infection. Total RNA extracted was subjected to qRT-PCR analysis by the 2^-ΔΔCt method^ with an 18S ribosomal RNA was used for normalization. Relative mRNA expression to the day 6 post MCV_WT_-infected sample is shown. Error bars indicate SD. **(B)** Establishment of BJ.hTERT TRE sT cells that inducibly express codon-optimized sT. BJ.hTERT cells stably transduced with TRE-sT lentivirus were treated with 0.5 μg/mL of doxycycline. MCV sT protein expression was detected by immunoblot using 2T2 antibody. Hsp70 was used as a loading control.
(TIF)

S4 Fig Subclassification of top 4 sT_WT_-associated protein classes identified by PANTHER. **(A)** Differential proximity-based biotinylation between sT_WT_-BirA and sT_LTSD_-BirA. Biotinylated proteins purified from 293 TRE Emp-BirA, sT_WT_-BirA, and sT_LTSD_-BirA cells were detected by streptavidin antibody. **(B)** Sub-classification of the four largest protein classes found by PANTHER analysis. Nucleic acid metabolism, cytoskeletal, metabolite interconversion enzymes, and membrane traffic protein classes account for over 50% of all detected proteins. Sub-classification allows higher specificity of protein function to improve identification of potential interactors. (C) To Identify potential background for interactors in Fig 6E, we exploited the CRAPome database for background spectral counts detected in previous studies. We took the spectral counts from our Bio-ID and compared them to the overall average spectral counts from previous experiments and determined a fold difference between them. We saw >1.7 fold increase for all of our potential interactors except MRTFA and MRTFB in Fig 6E.
(TIF)

S5 Fig Validation of sT interaction with transcriptional regulators and co-localization in LT replication foci. **(A)** Both N- and C-terminally tagged sT interact with Cux1, c-Jun, CBP, and BRD9. Lysates from 293 cells expressing NSF-sT_WT_ and CSF-sT_WT_ were subjected to immunoprecipitation with FLAG antibody, and immunoprecipitants were immunoblotted with FLAG antibodies. 2.5% input lysates were loaded on the same gel with immunoprecipitants. **(B)** Nuclear pixel matching co-localization analysis for sT and either Cux1, c-Jun, or CBP1 in the nucleus of NSF-sT_WT_ or NSF-sT_LTSD_-positive cells. Pearson correlation coefficient (r) was determined within a specific 0.22 μm slice of the selected nucleus showing the clearest protein signal. One representative cell used in the analyses is shown with a red region of interest (ROI) border. **(C)** r was determined by pixel matching analyses from >3 cells for Cux1, c-Jun, or CBP1 co-localization analyses (Olympus). Error bar indicates SD. **(D)** Co-localization of LT replication foci with Cux1 and c-Jun, but not with CBP1. Confocal immunofluorescence images of MCVΔsT-transfected 293 TRE sT_WT_ and sT_LTSD_ cells co-stained with anti-MCV LT (green) and either anti-Cux1, anti-c-Jun, or anti-CBP (red). White line in DAPI image represents 10 μm.
(TIF)

S6 Fig Confirmation of siRNA knockdown on target proteins and effect of small molecule inhibitors on viral MCV origin replication. **(A)** Confirmation of siRNA knockdown activity used in Fig 8. A control siRNA (siCtrl) and two of each siRNA targeting either c-Jun (sic-Jun), BRD9 (siBRD9), Cux1 (siCux1), or CBP (siCBP) were transfected in 293 cells, and protein expression of target proteins was examined by immunoblots. Hsp70 was detected as a loading control. Numbers on each blot indicate relative protein expression to siCtrl quantitated by LI-COR immunoblots. Protein expression was normalized by Hsp70. **(B)** Neither CBP/P300 inhibition by A-485 nor BRD9 inhibition by I-BRD9 inhibits MCV origin replication induced by sT-mediated LT stabilization. 293 cells co-transfected with p339Ori, LT expression vector, pEGFP, and pcDNA MCV sT_WT_ or empty (Emp) were treated with various amounts of A-485 and I-BRD9 for 48 h before harvest at 72 h p.t.. Episomal DNA was extracted and treated with DpnI. By qPCR, replication of p339Ori was determined using the 2^-ΔΔCt^ method with pEGFP as a transfection efficiency control. Error bars indicate SD. Unpaired T-test was used for statistical analysis. Significance was determined in comparison to mock-treated sT_WT_. *, P<0.05; **, P<0.01; ***, P<0.001. **(C)** LT protein expression was determined by immunoblots to confirm LT stabilization by sT for origin replication assay. Hsp70 was used as a loading control.
(TIF)

S7 Fig Role of EP400/Tip60 in MCV replication and transcription. **(A)** LTSD, but not the EP400-binding property of MCV sT, controls MCV origin replication. For MCV replicon assay, 293 cells co-transfected with p339Ori, LT expression vector, various sT expression vectors, and pEGFP were harvested at 48 h p.t.. MCV replicon assay was performed as described in S6B Fig. Immunoblots were performed on the same lysates to confirm LT protein stabilization by sT_WT_, sT_83-88A_, and sT_4M_. However, LT stabilization did not occur with three LTSD mutants including sT_90-95A_, which was previously defined as an EP400 binding mutant. **(B)** EP400 mutants, except for the sT_90-95A_ mutant, rescue MCVΔsT replication in 293 cells. MCVΔsT was co-transfected with sT_WT_, sT_L142A_ PP2A-binding mutant, sT_LTSD_ mutant, and three EP400-binding mutants (sT_90-95A_, sT_83-88A_, sT_4M_) in 293 cells. Samples were harvested at day 4 p.t. and analyzed by Southern hybridization with an MCV probe (upper panel) and immunoblots to detect LT and VP1 protein expression (bottom panel). **(C)** Effect of EP400 binding mutations in the MCV genome on viral DNA replication. 293 cells were transfected with MCT_WT_, MCVΔsT, MCV_rep-_, and three MCV mutants that ablate EP400 binding (MCV.sT_90-95A_, MCV.sT_83-88A_ and MCV.sT_4M_) and harvested at day 4 p.t. for Southern blot and qPCR were performed as described in Fig 3E. **(D)** A knockdown of EP400 with multiple shRNA’s increases early and late gene viral transcription in 293 cells. 293 cells were infected with control (shCtrl) and three EP400-targeting lentiviral shRNAs (shEP400.1, shEP400.2, and shEP400.3 (S3 Table)) and transfected with MCV_WT_ at day 1 post-lentiviral infection. Cells were harvested at day 5 for qRT-PCR analysis for MCV early (blue) and late (red) gene expression, detected by PanT and VP2 (set 2) primer pairs, respectively. Relative MCV gene expression was determined by the 2^-ΔΔCt^ method with 18S ribosomal RNA as a control. **(E)** Confirmation of EP400 knockdown by qRT-PCR for samples used in **(D)**.
(TIF)

S1 Dataset sT_WT_- and sT_LTSD_-proximal host proteins identified by Bio-ID. (XLSX)

S1 Methods MCV replicon assays and EP400 knockdown. (DOCX)

S1 Table Primers used for quantitative PCR analysis. (PDF)

S2 Table Mutagenesis primers. (PDF)

S3 Table Oligonucleotides for shRNA construction. (PDF)
