# Supplementary figures and images for: Merkel cell polyomavirus small T antigen is a viral transcription activator that is essential for viral genome maintenance
Source: PLoS Pathog. 2022 Dec 27;18(12):e1011039. doi: 10.1371/journal.ppat.1011039 (PMC9829177; doi:10.1371/journal.ppat.1011039)

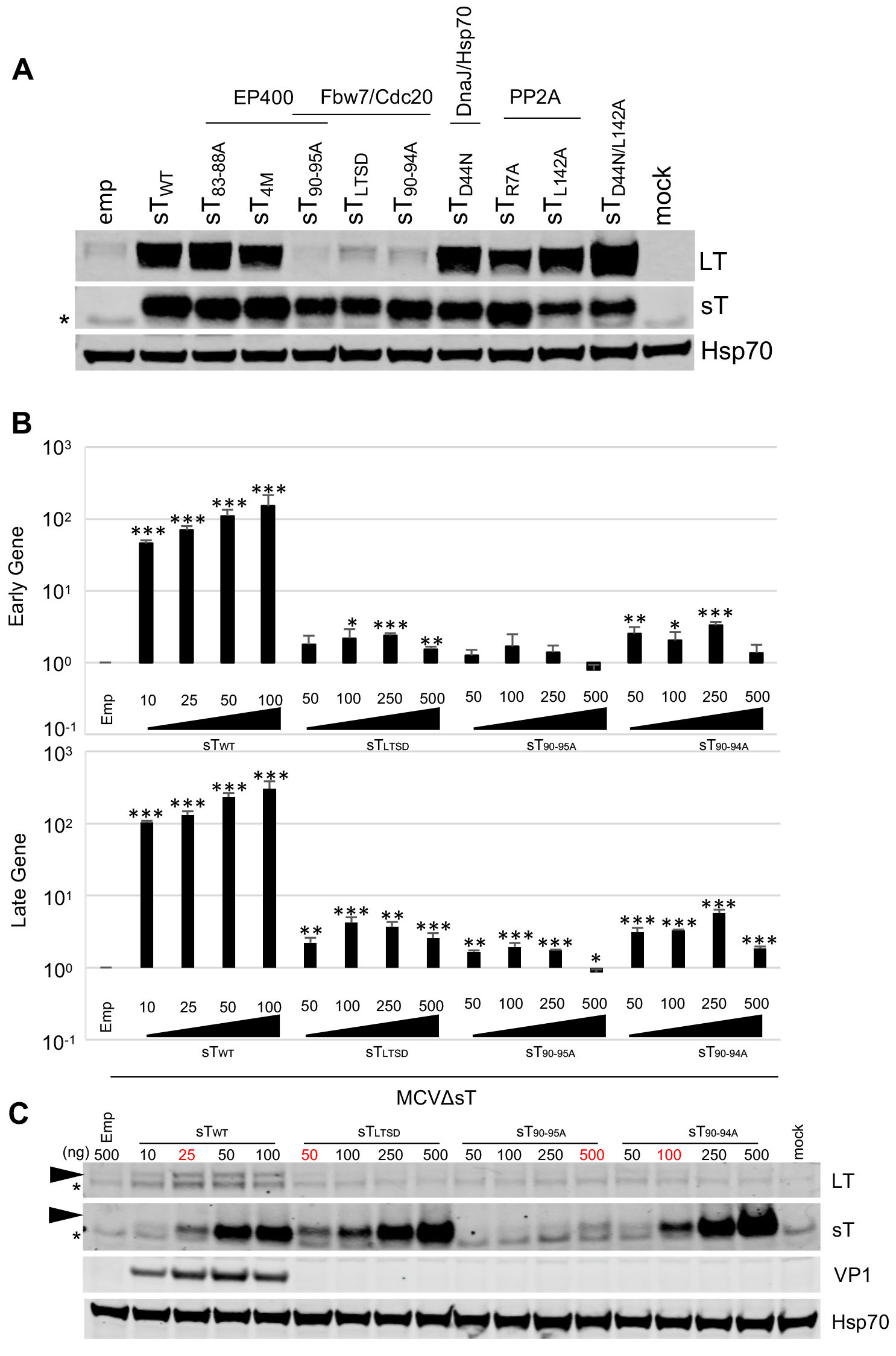

Supplement: S1 Fig — (A) Stabilization of MCV LT by co-expression of sTWT and various sT mutants. 293 cells co-transfected with LT and various MCV sT mutant expression vectors were harvested at 48 h p.t. and cell lysates were subjected to immunoblots with LT (CM2B4) and MCV sT (2T2) antibodies. Hsp70 was used to show equal protein loading. Three mutants, sTLTSD, sT90-95A, sT90-94A, that have mutations surrounding the LTSD (aa 90–95) failed to increase LT expression. While sT90-95A is one of three EP400 binding mutants [20], two other mutants, sT83-88A and sT4M, stabilized LT. MCV sT harboring mutations in DnaJ (D44N), PP2A (R7A and L142A), and both DnaJ and PP2A (D44N/L142A) increased LT to wild type levels. (B) 293 TRE-sT cells were co-transfected with MCVΔsT and pcDNA empty (Emp), and increasing concentrations of pcDNA sTWT or LTSD mutants (sTLTSD, sT90-95A, and sT90-94A). Samples were harvested on day 4 p.t. RNA was extracted and converted to cDNA for qRT-PCR analysis to determine early and late gene expression as described in Fig 2B legend. Error bars represent SD. Regardless of concentration, LTSD mutants could not induce expression of the early or late gene as sTWT does. (C) Replicated samples prepared as in S1B Fig were extracted for protein and used to determine sT, LT, and VP1 protein expression by immunoblot. Hsp70 was used as a loading control. Comparable protein expression is in red text. Even the smallest concentration of sTWT, 10 ng, can express LT and VP1, while the LTSD mutants cannot rescue LT or VP1, no matter their concentration. (TIF) [file ppat.1011039.s001.tif]

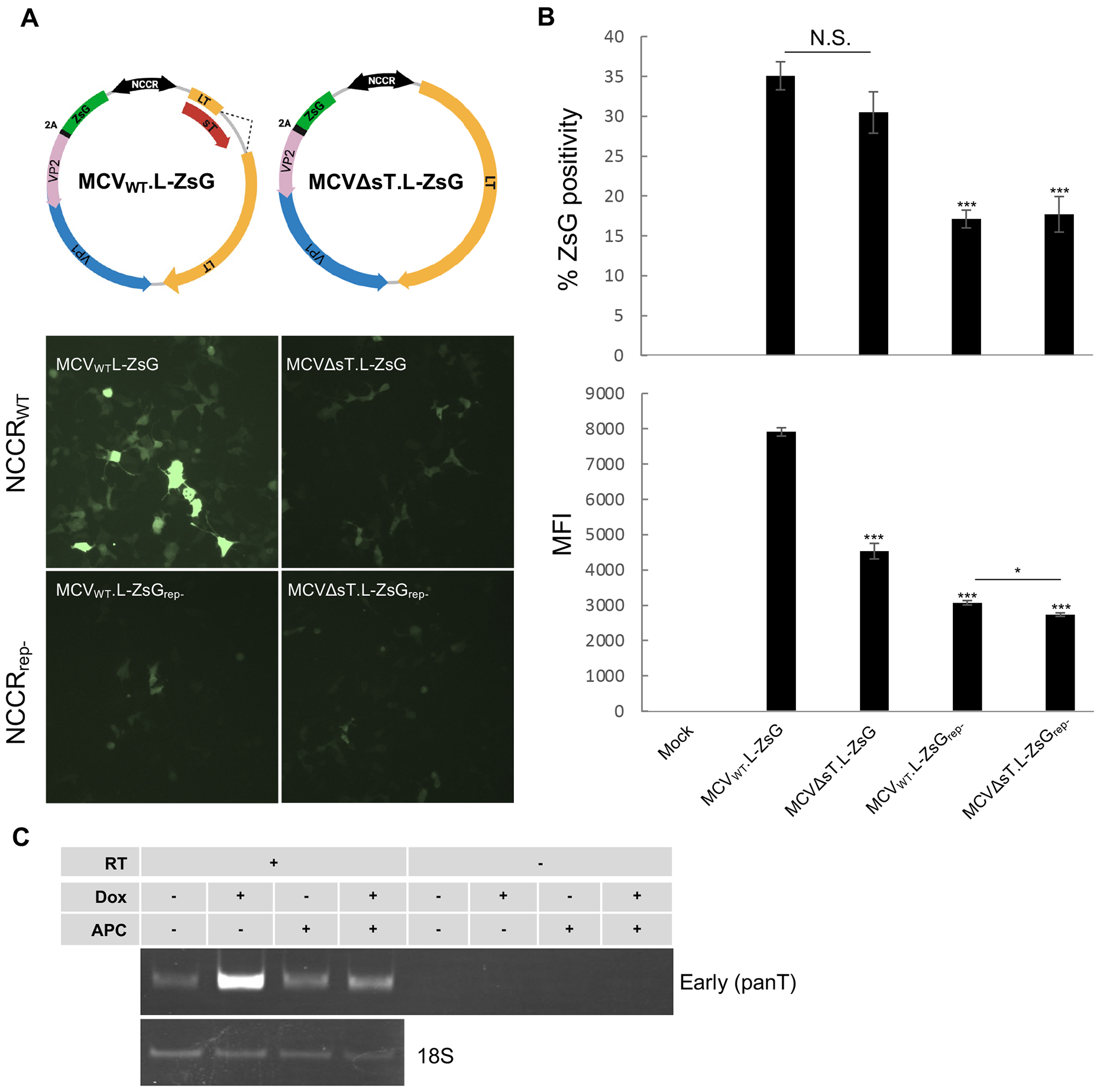

Supplement: S2 Fig — (A) Generation of MCV late gene reporter viruses with or without sT deletion. MCVWT.L-ZsG and MCVΔsT.L-ZsG encoding the ZsGreen (ZsG)-FMDV-2A sequence before the VP2 coding sequence. 293 cells transfected with MCVWT.L-ZsG and MCVΔsT.L-ZsG with or without the rep- mutation were imaged under an inverted microscope at day 4 p.t. (B) Quantification of late gene-driven ZsG positive cells (top panel) and mean fluorescence intensity (bottom panel). Cells obtained from 6 independent transfections were analyzed by flow cytometry. sT deletion does not significantly decrease ZsG positive cells whereas introducing a mutation into the NCCR (MCVΔsT.L-ZsGrep-) decreased percent positivity by 50%. On the other hand, sT deletion reduced mean fluorescence intensity (MFI) by 43%. However, the rep- mutation cancelled the sT deletion effect and further attenuated MFI. Based on the decrease in ZsG positivity by the rep- mutation, ZsG positivity appears to be under the control of viral replication. In contrast, MFI may reflect late promoter activity since per-cell-intensity of ZsG was similarly lower in cells transfected with MCVΔsT.L-ZsG, MCVWT.L-ZsGrep-, and MCVΔsT.L-ZsGrep- as in S2A Fig. (C) Day 4 p.t. samples treated with or without APC from Fig 4C and 4D were used to examine early gene mRNA expression. Total RNA was extracted and converted to cDNA using the Super Script IV and the PanT2 primer in S1 Table. PCR was performed using the same PanT primers as in Fig 4D. 18S ribosomal RNA was amplified from cDNA that was generated by iScript as a control. (TIF) [file ppat.1011039.s002.tif]

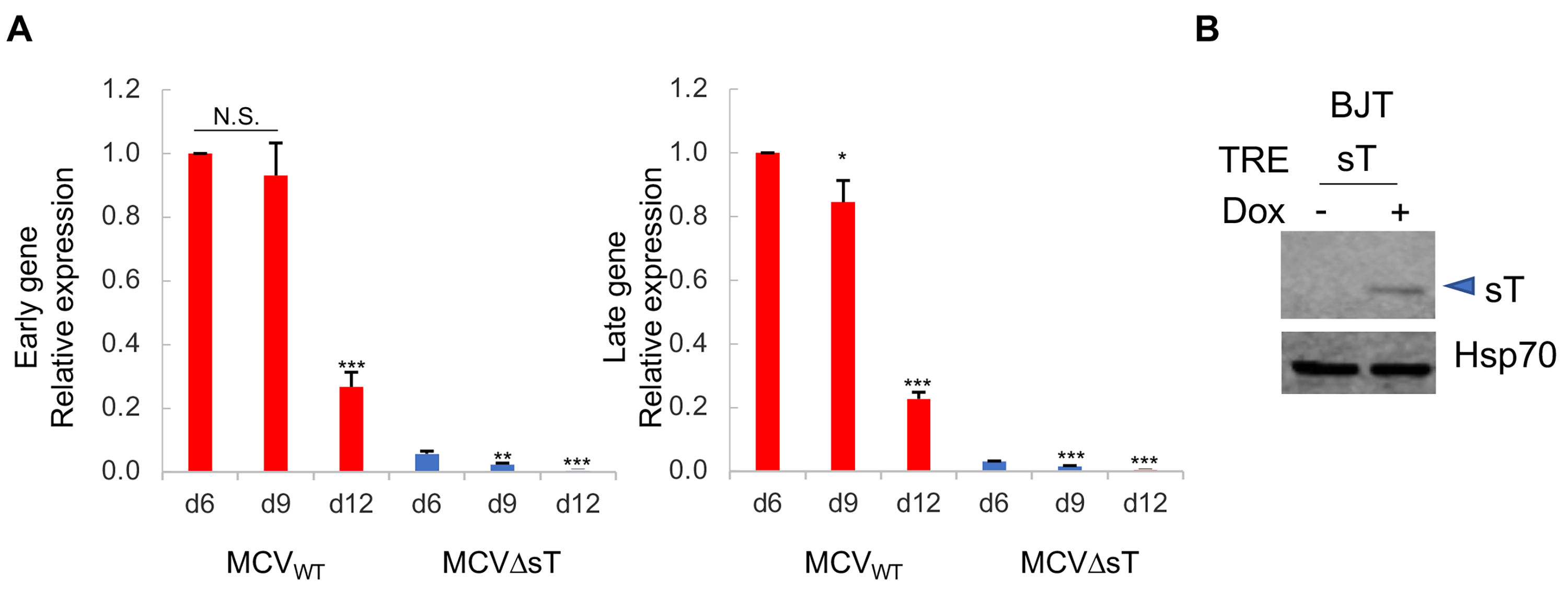

Supplement: S3 Fig — (A) sT regulates VP1 expression in infected permissive cells. sT deletion in the MCV genome reduces early and late viral gene expression. BJ.hTERT cells infected with MCVWT and MCVΔsT were harvested at various time points after infection. Total RNA extracted was subjected to qRT-PCR analysis by the 2-ΔΔCt method with an 18S ribosomal RNA was used for normalization. Relative mRNA expression to the day 6 post MCVWT-infected sample is shown. Error bars indicate SD. (B) Establishment of BJ.hTERT TRE sT cells that inducibly express codon-optimized sT. BJ.hTERT cells stably transduced with TRE-sT lentivirus were treated with 0.5 μg/mL of doxycycline. MCV sT protein expression was detected by immunoblot using 2T2 antibody. Hsp70 was used as a loading control. (TIF) [file ppat.1011039.s003.tif]

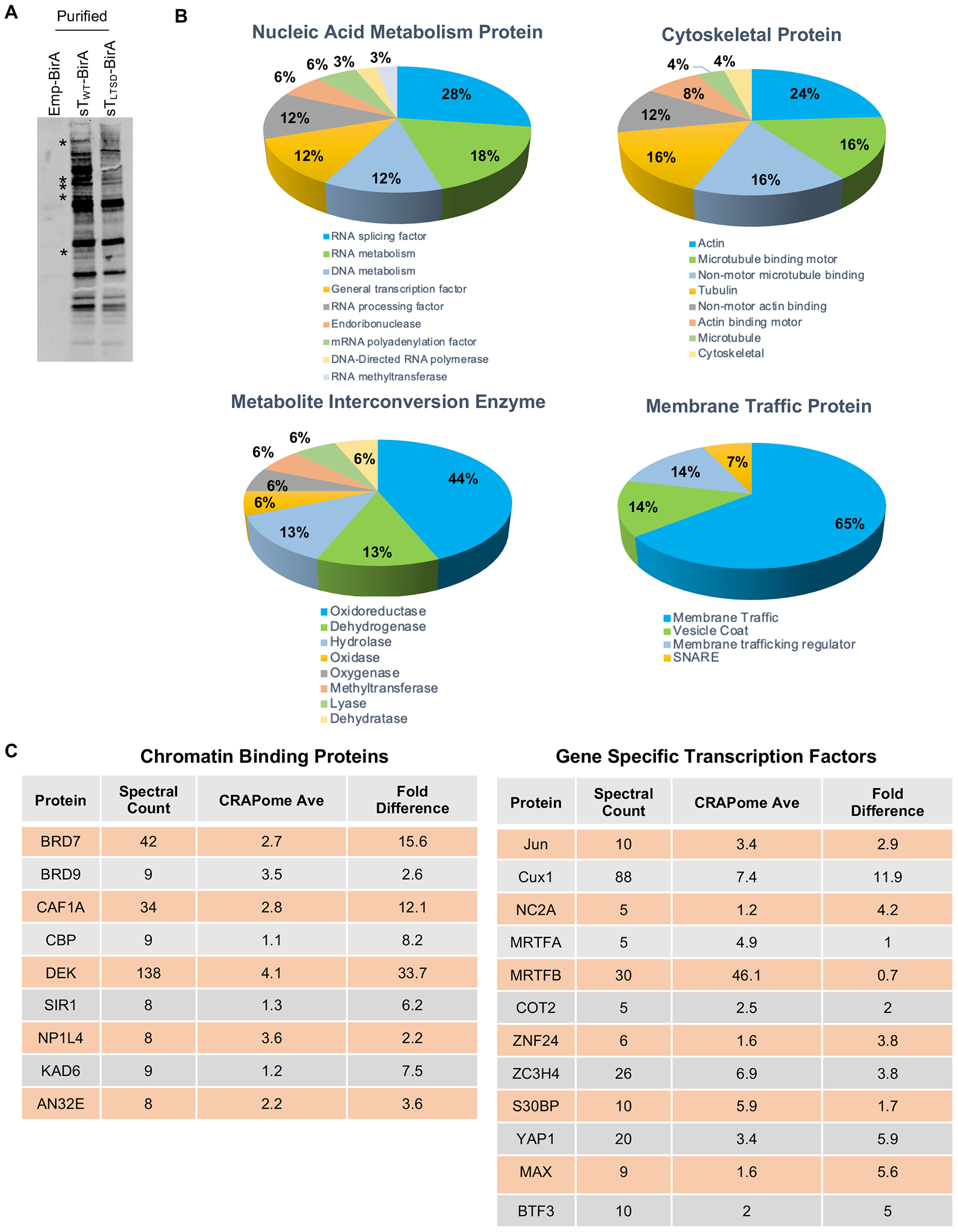

Supplement: S4 Fig — (A) Differential proximity-based biotinylation between sTWT-BirA and sTLTSD-BirA. Biotinylated proteins purified from 293 TRE Emp-BirA, sTWT-BirA, and sTLTSD-BirA cells were detected by streptavidin antibody. (B) Sub-classification of the four largest protein classes found by PANTHER analysis. Nucleic acid metabolism, cytoskeletal, metabolite interconversion enzymes, and membrane traffic protein classes account for over 50% of all detected proteins. Sub-classification allows higher specificity of protein function to improve identification of potential interactors. (C) To Identify potential background for interactors in Fig 6E, we exploited the CRAPome database for background spectral counts detected in previous studies. We took the spectral counts from our Bio-ID and compared them to the overall average spectral counts from previous experiments and determined a fold difference between them. We saw >1.7 fold increase for all of our potential interactors except MRTFA and MRTFB in Fig 6E. (TIF) [file ppat.1011039.s004.tif]

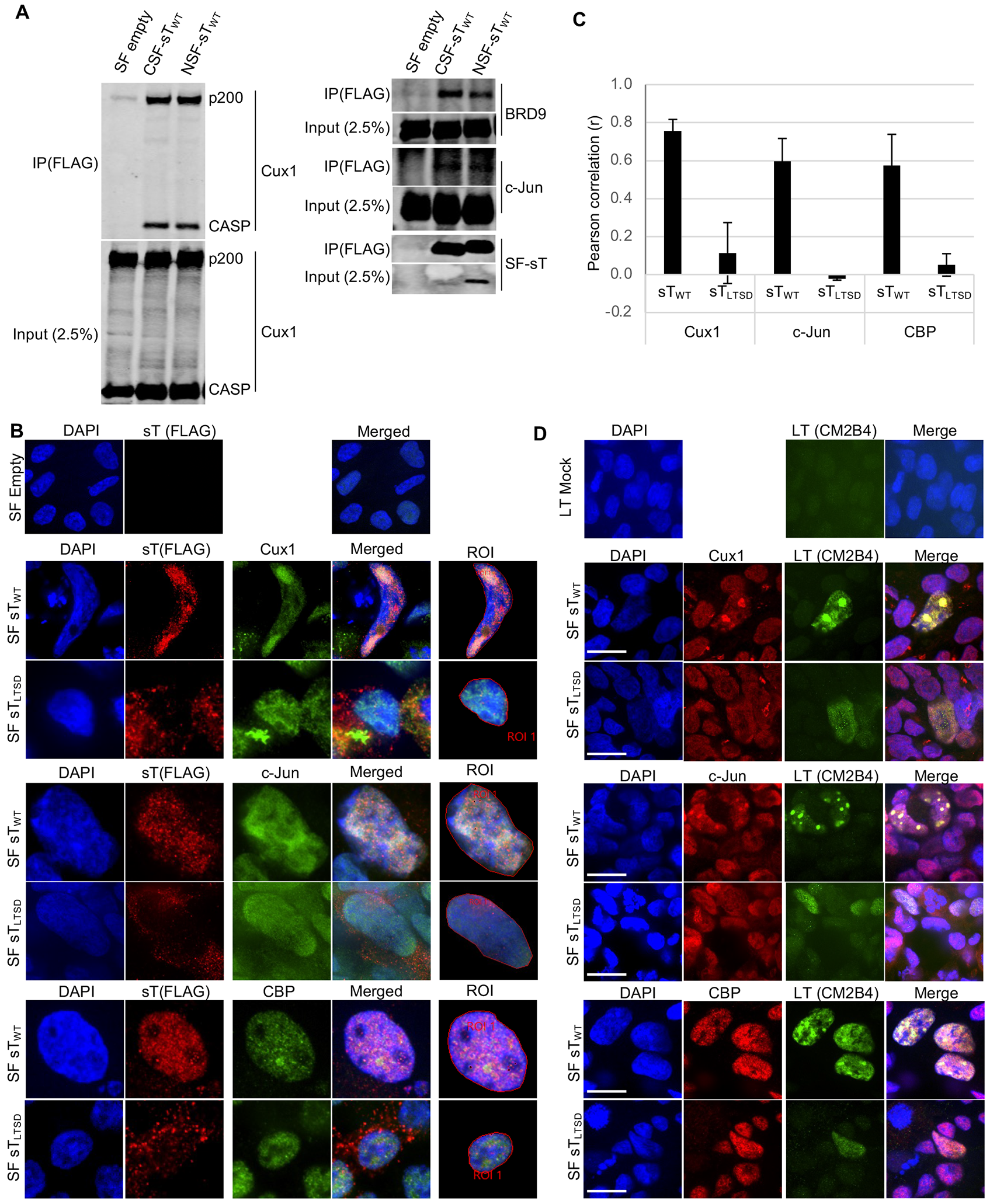

Supplement: S5 Fig — (A) Both N- and C-terminally tagged sT interact with Cux1, c-Jun, CBP, and BRD9. Lysates from 293 cells expressing NSF-sTWT and CSF-sTWT were subjected to immunoprecipitation with FLAG antibody, and immunoprecipitants were immunoblotted with FLAG antibodies. 2.5% input lysates were loaded on the same gel with immunoprecipitants. (B) Nuclear pixel matching co-localization analysis for sT and either Cux1, c-Jun, or CBP1 in the nucleus of NSF-sTWT or NSF-sTLTSD-positive cells. Pearson correlation coefficient (r) was determined within a specific 0.22 μm slice of the selected nucleus showing the clearest protein signal. One representative cell used in the analyses is shown with a red region of interest (ROI) border. (C) r was determined by pixel matching analyses from >3 cells for Cux1, c-Jun, or CBP1 co-localization analyses (Olympus). Error bar indicates SD. (D) Co-localization of LT replication foci with Cux1 and c-Jun, but not with CBP1. Confocal immunofluorescence images of MCVΔsT-transfected 293 TRE sTWT and sTLTSD cells co-stained with anti-MCV LT (green) and either anti-Cux1, anti-c-Jun, or anti-CBP (red). White line in DAPI image represents 10 μm. (TIF) [file ppat.1011039.s005.tif]

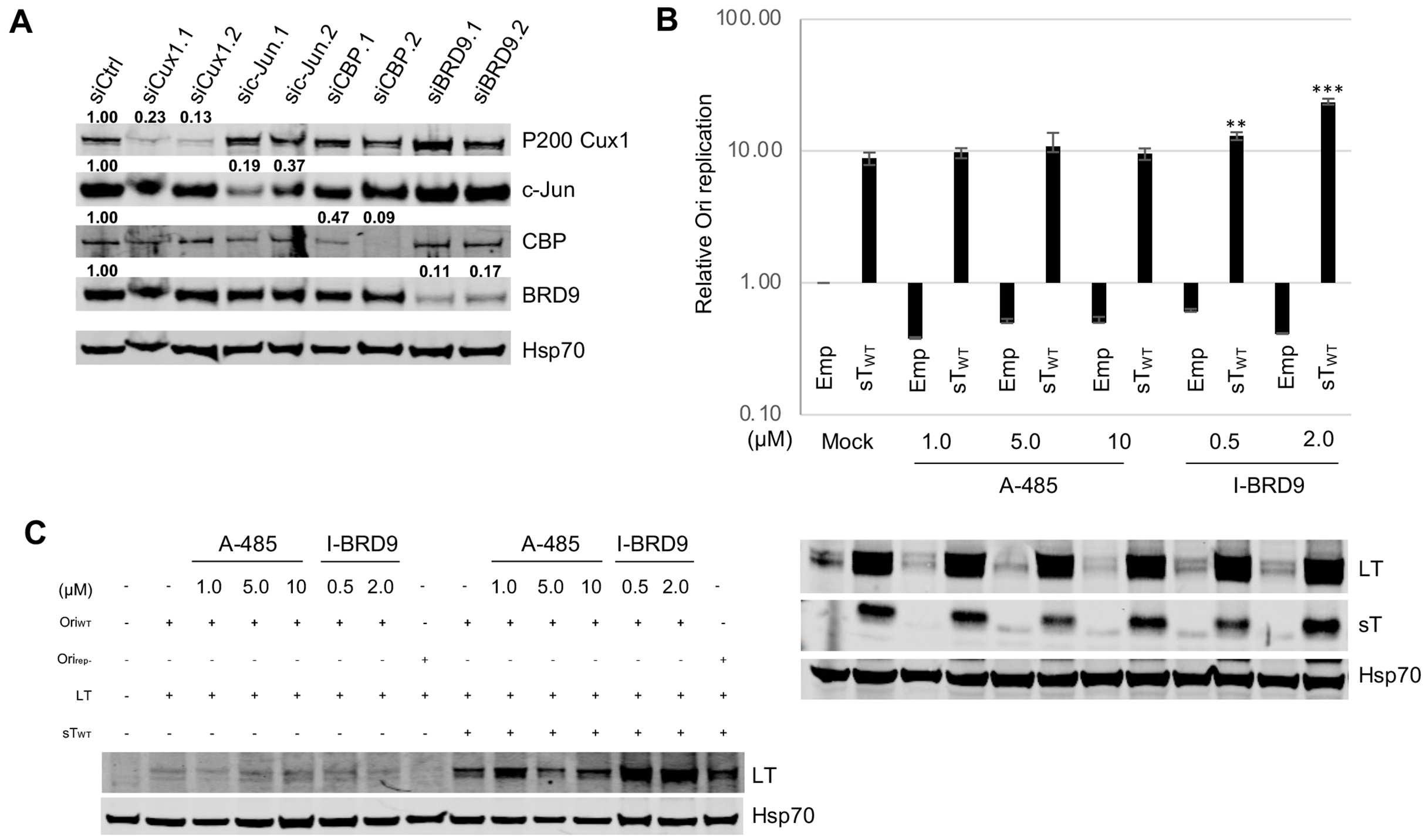

Supplement: S6 Fig — (A) Confirmation of siRNA knockdown activity used in Fig 8. A control siRNA (siCtrl) and two of each siRNA targeting either c-Jun (sic-Jun), BRD9 (siBRD9), Cux1 (siCux1), or CBP (siCBP) were transfected in 293 cells, and protein expression of target proteins was examined by immunoblots. Hsp70 was detected as a loading control. Numbers on each blot indicate relative protein expression to siCtrl quantitated by LI-COR immunoblots. Protein expression was normalized by Hsp70. (B) Neither CBP/P300 inhibition by A-485 nor BRD9 inhibition by I-BRD9 inhibits MCV origin replication induced by sT-mediated LT stabilization. 293 cells co-transfected with p339Ori, LT expression vector, pEGFP, and pcDNA MCV sTWT or empty (Emp) were treated with various amounts of A-485 and I-BRD9 for 48 h before harvest at 72 h p.t.. Episomal DNA was extracted and treated with DpnI. By qPCR, replication of p339Ori was determined using the 2-ΔΔCt method with pEGFP as a transfection efficiency control. Error bars indicate SD. Unpaired T-test was used for statistical analysis. Significance was determined in comparison to mock-treated sTWT. *, P<0.05; **, P<0.01; ***, P<0.001. (C) LT protein expression was determined by immunoblots to confirm LT stabilization by sT for origin replication assay. Hsp70 was used as a loading control. (TIF) [file ppat.1011039.s006.tif]

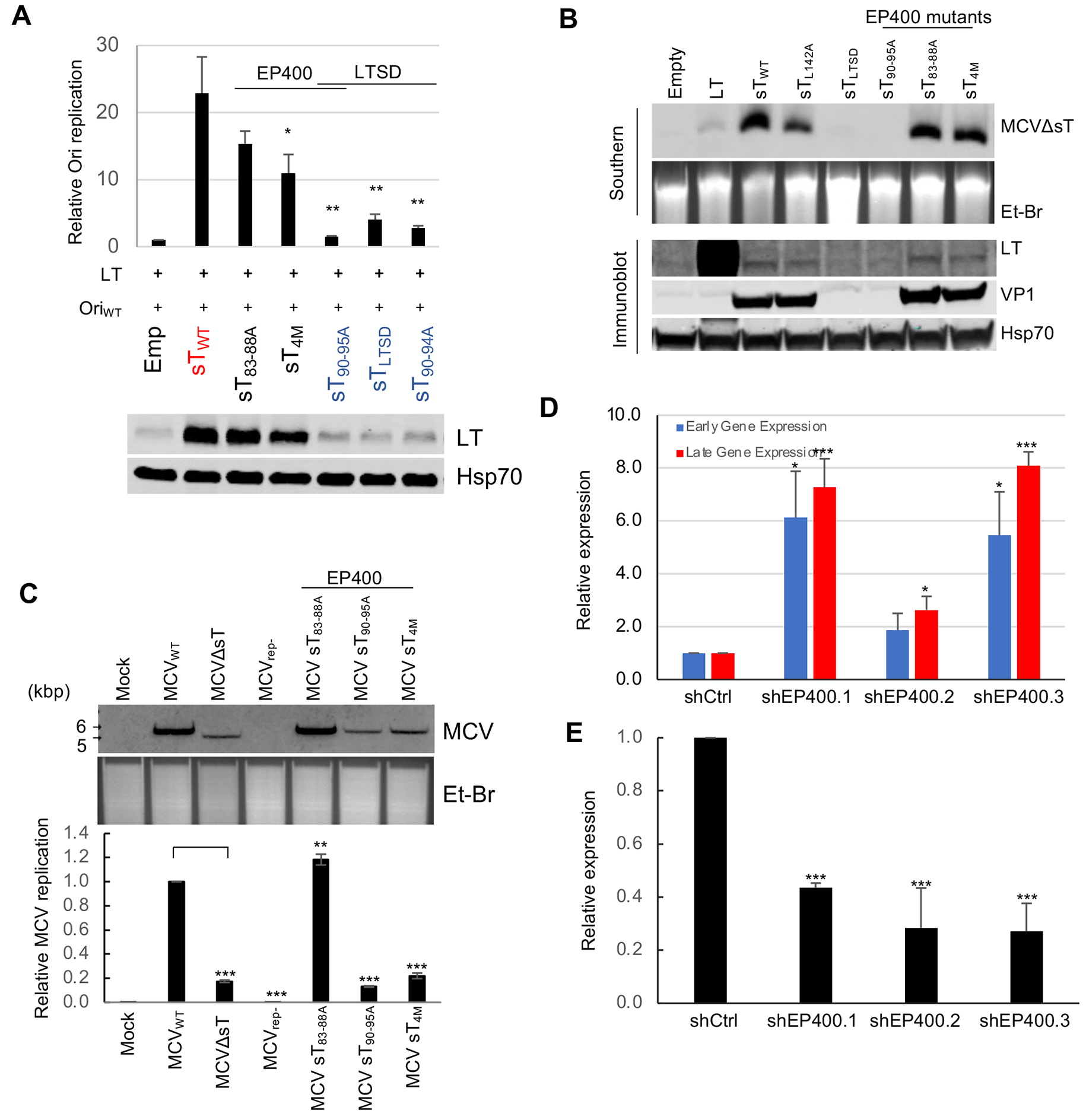

Supplement: S7 Fig — (A) LTSD, but not the EP400-binding property of MCV sT, controls MCV origin replication. For MCV replicon assay, 293 cells co-transfected with p339Ori, LT expression vector, various sT expression vectors, and pEGFP were harvested at 48 h p.t.. MCV replicon assay was performed as described in S6B Fig. Immunoblots were performed on the same lysates to confirm LT protein stabilization by sTWT, sT83-88A, and sT4M. However, LT stabilization did not occur with three LTSD mutants including sT90-95A, which was previously defined as an EP400 binding mutant. (B) EP400 mutants, except for the sT90-95A mutant, rescue MCVΔsT replication in 293 cells. MCVΔsT was co-transfected with sTWT, sTL142A PP2A-binding mutant, sTLTSD mutant, and three EP400-binding mutants (sT90-95A, sT83-88A, sT4M) in 293 cells. Samples were harvested at day 4 p.t. and analyzed by Southern hybridization with an MCV probe (upper panel) and immunoblots to detect LT and VP1 protein expression (bottom panel). (C) Effect of EP400 binding mutations in the MCV genome on viral DNA replication. 293 cells were transfected with MCTWT, MCVΔsT, MCVrep-, and three MCV mutants that ablate EP400 binding (MCV.sT90-95A, MCV.sT83-88A and MCV.sT4M) and harvested at day 4 p.t. for Southern blot and qPCR were performed as described in Fig 3E. (D) A knockdown of EP400 with multiple shRNA’s increases early and late gene viral transcription in 293 cells. 293 cells were infected with control (shCtrl) and three EP400-targeting lentiviral shRNAs (shEP400.1, shEP400.2, and shEP400.3 (S3 Table)) and transfected with MCVWT at day 1 post-lentiviral infection. Cells were harvested at day 5 for qRT-PCR analysis for MCV early (blue) and late (red) gene expression, detected by PanT and VP2 (set 2) primer pairs, respectively. Relative MCV gene expression was determined by the 2-ΔΔCt method with 18S ribosomal RNA as a control. (E) Confirmation of EP400 knockdown by qRT-PCR for samples used in (D). (TIF) [file ppat.1011039.s007.tif]
