# Supplementary material for: Merkel cell polyomavirus small T antigen is a viral transcription activator that is essential for viral genome maintenance
Source: PLoS Pathog. 2022 Dec 27;18(12):e1011039. doi: 10.1371/journal.ppat.1011039 (PMC9829177; doi:10.1371/journal.ppat.1011039)
Supplement: S1 Methods — (DOCX) [file ppat.1011039.s009.docx]

**S1 Methods**

**MCV replicon (origin replication) assay**

Parental 293 cells were seeded in 6 well plates at approximately 50% cell density. 24 h after seeding, cells were transfected. The transfection included 400 ng of pcDNA6 MCV LT, 400 ng of pcDNA6 sT_WT_ or other sT mutants, 400 ng of pCR Ori339 or pCR Ori350, and 100 ng of pEGFP. pCR Ori350 is a replication defective mutant that involves a singular mutation in the NCCR region which eliminates the ability of LT binding. To normalize transfection between each well, the EGFP plasmid was transfected into each well. 48 h p.t., cells were harvested for episomal DNA extraction and assessed using qPCR.

**Construction and infection of EP400-targeting lentiviral shRNA**

For EP400 lentiviral shRNA construction, we used pENTR1A e7SK-Pro vector generated previously [1,2]. Three paired sense and anti-sense oligonucleotides were annealed to generate shRNA-coding sequences (shEp400.1, shEP400.2, and shEP400.3) that target EP400 mRNA (**S3 Table)** and ligated into pENTR1A e7SK-Pro construct by *AgeI* and *EcoRI* restriction sites. The shRNA expression cassette cloned in entry vectors was recombined into pMuLE Lenti Dest-puro. Control shRNA (shCtrl) construction was previously described [1]. For lentivirus production, lentiviral constructs expressing three distinct shEP400 was co-transfected with psPAX2 (Addgene plasmid# 12260) and pMD2.G (Addgene plasmid# 12259) into 293FT cells by Lipofectamine 2000 (Invitrogen). At 16h of transfection, cell culture medium containing plasmid DNAs and transfection reagent was removed, and fresh 10% FBS DMEM was added. At 72 h post transfection, culture supernatant was harvested, and the lentivirus was aliquoted into small tubes. Five hundred μL of the lentivirus was infected into MCV-positive MCC cells in the presence of 1 μg/mL polybrene for 2 days, and then infected cells were selected with puromycin (1μg/mL) for 4 days. To quantitate EP400 knockdown efficiency, qRT-PCR was performed by using primers listed in **S1 Table**.

References

1. Velasquez C, Amako Y, Harold A, Toptan T, Chang Y, Shuda M. Characterization of a Merkel Cell Polyomavirus-Positive Merkel Cell Carcinoma Cell Line CVG-1. Front Microbiol. 2018;9:713. Epub 2018/04/27. doi: 10.3389/fmicb.2018.00713. PubMed PMID: 29696010; PubMed Central PMCID: PMCPMC5905237.

2. Harold A, Amako Y, Hachisuka J, Bai Y, Li MY, Kubat L, et al. Conversion of Sox2-dependent Merkel cell carcinoma to a differentiated neuron-like phenotype by T antigen inhibition. Proc Natl Acad Sci U S A. 2019. Epub 2019/09/19. doi: 10.1073/pnas.1907154116. PubMed PMID: 31527246.
