# Supplementary material for: Merkel cell polyomavirus small T antigen is a viral transcription activator that is essential for viral genome maintenance
Source: PLoS Pathog. 2022 Dec 27;18(12):e1011039. doi: 10.1371/journal.ppat.1011039 (PMC9829177; doi:10.1371/journal.ppat.1011039)
Supplement: S1 Table — (PDF) [file ppat.1011039.s010.pdf]

S1 Table

qPCR Primer	5' - Sense - 3'	5' - AntiSense - 3'
**PanT**	GCTCCTAATTGTTATGGCAACAT	GCTCCAAAGGGTGTTCAATTC
**PanT2**	CACTTCTGAGCTTGTGGATATTT	
**VP2 set 1**	GAGGCATGCACTTATGGCCT	TCTGAAAGACCCACCGGCTA
**VP2 set 2**	GAGGGATGTTTCGTGGGTAGG	GCCTCTCCTGATAAAAGGCCC
**18S**	GGACACGGACAGGATTGACA	ACCCACGGAATCGAGAAAGA
**GAPDH**	GTTTACATGTTCCAATATGATTCC	TCCTGGAAGATGGTGATGG
**Rep**	TGGATGGCTTTCTTGCCG	GAGAACCTGCGTGCAATC
**eGFP**	ACGTAAACGGCCACAAGTTC	AAGTCGTGCTGCTTCATGTG
**EP400**	CCAGGAGAGGGAAAGAATTGAG	TTCATCGTCCACTTCGTCATC
