# Supplementary material for: Merkel cell polyomavirus small T antigen is a viral transcription activator that is essential for viral genome maintenance
Source: PLoS Pathog. 2022 Dec 27;18(12):e1011039. doi: 10.1371/journal.ppat.1011039 (PMC9829177; doi:10.1371/journal.ppat.1011039)
Supplement: S2 Table — (PDF) [file ppat.1011039.s011.pdf]

S2 Table

Mutagenesis Primer	5' - Sequence - 3'
**MCV.ΔsT.F**	GACTTCTCTATGTTTGATGAGGTTGACGAGGCCCCTATATATGG
**MCV.ΔsT.R**	CATATATAGGGGCCTCGTCAACCTCATCAAACATAGAGAAGTC
**MCV.sT83-88A.F**	AAAGCTGCCGCTGCCGCTGCCGGAACTTTAAAGGATTATATGCAAAGTGGATATAATG
**MCV.sT83-88A.R**	TCCGCCAGCGGCAGCGGCAGCTTTTGTACTGACCTCATCAAACATAGAGAAG
**MCV.sT4M.F**	TTTCCTTGGAGCAGTTATGGCACTTTAAGCAGTTATATGCAAAGTGGATATAATGCTAGATTTTGCAG
**MCV.sT4M.R**	TTTGCATATAACTGCTTAAAGTTCCATAACTGCTCCAAGGAAATTTTGTACTGACCTCATC
**MCV.sT90-94A.F**	GGAGCTGCCGCTGCCGCTATGCAAAGTGGATATAATGCTAGATTTTGCAGAG
**MCV.sT90-94A.R**	TTGCATAGCGGCAGCGGCAGCTCCATATTCTTCCCAAGGAAATTTTGTACTG
**MCV.sTA90-95.F**	GGAGCTGCCGCTGCCGCTGCCCACAAGTGGATATAATGCTAGATTTTGCAGAG
**MCV.sTA90-95.R**	TTGGGCAGCGGCAGCGGCAGCTCCATATTCTTCCCAAGGAAATTTTGTACT
**MCV.sTA91-95.F**	GGAACTGCCGCTGCCGCTGCCCAAAGTGGATATAATGCTAGATTTTGCAGAG
**MCV.sTA91-95.R**	TTGGGCAGCGGCAGCGGCAGTTCCATATTCTTCCCAAGGAAATTTTGTACTG
**sTco.83-88A.F**	AAGGCCGCAGCCGCAGCCGCAGGCACACTCAAAGATTACATGCAG
**STco.83-88A.R**	TGCGGCTGCGGCTGCGGCCTTGGTAGACACCTCATCAAACATGG
**sTco.4M.F**	CCCTGGAGCAGCTATGGCACACTCAGCAGCTACATGCAGAGTGGCTAC
**sTco.4M.R**	ATGTAGCTGCTGAGTGTGCCATAGCTGCTCCAGGGGAACTTGGTAGAC
**sTco.90-94A.F**	GGCGCCGCAGCCGCAGCCATGCAGAGTGGCTACAATGCTAG
**sTco.90-94A.R**	CTGTGCGGCTGCGGCTGCGGCGCCATATTCCTCCCAGGGGAAC
**sTco.90-95A.F**	GGCGCCGCAGCCGCACAGAGTGGCTACAATGCTAGATTCTG
**sTco.90-95A.R**	CTGTGCGGCTGCGGCTGCGGCGCCATATTCCTCCCAGGGGAAC
**AvrII.MCV.NCR.F**	GCTGCCTAGGTGACTTTTTTTTTTCAAGTTGGC
**AvrII.MCV.NCR.R**	GTCACCTAGGCAGCCAAGTTGTGGTTACATG
**BamHI.R**	CTACTGGATCCAGAGGATGAGGTG
**2A.VP2.F**	CGTCGAGTCCAACCCAGGGCCCATGGGGGGCATCATCACACTG
**2A.VP2.R**	CAGTGTGATGCCCCCCATGGGCCCTGGGTTGGACTCGACG
**VP2.NCR.ZsGreen.F**	GACATTAAAAGAGTAAGTATCCTTATTTATTTTTCAGGATGGCCCAGTCCAAGCACGGCC
**VP2.NCR.R**	GGCCGTGCTTGGACTGGGCCATCCTGAAAAATAAATAAGGATACTTACTCTTTTAATGTC
**VP2.PacI.R**	GAACATTAATTAAAGAACTTATTCCAACTACTTCTG
**KpnI.KozMCVsTco.F**	GGGCGGCGGTACCACCATGGACTTGGTCCTTAAC
**Not.delTCMCVsTco.R**	GGGCGGCGGCCGCGAAGAGATGCAAGTGAAGCAAGC
**Nhe.delICMCVsTco.F**	GGGCGGCTAGCGACTTGGTCCTTAACAGGAAAG
**Xho.StopSTCodOpt.R**	GGGCGGCTCGAGTTATCAGAAGAGATGCAAGTGAAGCAAGC
**AfeI.sTco.F**	GGGCGGCAGCGCTATGGACTTGGTCCTTAACAGGAAAGAACG
**KpnI.sTco.R**	GTGCGGTACCGAAGAGATGCAAGTGAAGC
**KpnI.linker.BirA.F**	CTGGGTACCGGCGGAGGCAGTGGCGGGGGTTCAGGTTCCAAGGACAACACCGTGCCCCTG
**BamHI.BirA.R**	GTCGGATCCTTATTTCTCGGCAGATCTC
