# Supplementary material for: Merkel cell polyomavirus small T antigen is a viral transcription activator that is essential for viral genome maintenance
Source: PLoS Pathog. 2022 Dec 27;18(12):e1011039. doi: 10.1371/journal.ppat.1011039 (PMC9829177; doi:10.1371/journal.ppat.1011039)
Supplement: S3 Table — (PDF) [file ppat.1011039.s012.pdf]

S3 Table

Oligonucleotides	5' - Sense - 3'	5' - AntiSense - 3'
**shEP400.1**	CCGGGCAGAATGATTTGGACATTGATCAAGAGTCAATGTCCAAATCATTCTGCTTTTTG	AATTCAAAAAGCAGAATGATTTGGACATTGACTCTTGATCAATGTCCAAATCATTCTGC
**shEP400.2**	CCGGGCTGCGAAGAAGCTCGTTACATCAAGAGTCTAACGAGCTTCTTCGCAGCTTTTTG	AATTCAAAAAGCTGCGAAGAAGCTCGTTAGACTCTTGATCTAACGAGCTTCTTCGCAGC
**shEP400.3**	CCGGGCCTTATACGAGGACGTTATCTCAAGAGGATAACGTCCTCGTATAAGGCTTTTTG	AATTCAAAAAGCCTTATACGAGGACGTTATCCTCTTGAGATAACGTCCTCGTATAAGGC
